# Antibiotic-impregnated calcium sulfate for the treatment of pediatric hematogenous osteomyelitis

**DOI:** 10.1186/s12887-022-03791-4

**Published:** 2022-12-23

**Authors:** Rui Tao, Jian-qun Wu, Ji-wei Luo, Liang Hong, Chun-hao Zhou, Guo-yun Cheng, Cheng-he Qin

**Affiliations:** 1 Department of Orthopaedics, Southern Medical University Zengcheng Branch of Nanfang Hospital, No. 28 Chuangxin Avenue Yongning Street, Zengcheng District Guangzhou, 511340 People’s Republic of China; 2 Department of Bone and Joint, School ofMedicine, The First Affiliated Hospital, Southern University of Science and Technology, Shenzhen, 518055 People’s Republic of China; 3 Department of Orthopaedics and Traumatology, Southern Medical University Nanfang Hospital, No. 1838, Guangzhou Ave. North, Guangzhou, Guangdong 510515 People’s Republic of China; 4 Department of Orthopaedics and Traumatology, Second Clinical Medical School, Guangdong Second Provincial General Hospital, Southern Medical University, Guangzhou, 510317 People’s Republic of China

**Keywords:** Hematogenous osteomyelitis, Pediatric, Calcium sulfate, Local antibiotic

## Abstract

**Background:**

Antibiotic-impregnated calcium sulfate has excellent curative efficacy in chronic osteomyelitis. However, its curative efficacy in pediatric hematogenous osteomyelitis has not been sufficiently studied. The purpose of this study was to evaluate the curative effects of antibiotic-impregnated calcium sulfate in the treatment of pediatric hematogenous osteomyelitis.

**Methods:**

Overall, twenty-one pediatric patients with hematogenous osteomyelitis treated at our hospital between 2013 and 2018 were included for assessment. The clinical history, clinical manifestation, infection recurrence rate, sinus leakage, incision leakage, pathological fractures, bone growth and surgical procedures were analyzed.

**Results:**

The infection recurrence rate was 0% (0/21) at a minimum of 31 months (range 31 to 91 months) of follow-up. Postoperative incision leakage was found in one pediatric patient. Osteolysis was found in one pediatric patient. Acceleration of bone growth occurred in one pediatric patient. Retardation of bone growth occurred in one pediatric patient. Genu valgus deformity occurred in one pediatric patient.

**Conclusions:**

Although noninfectious complications occurred, the curative effect of antibiotic-impregnated calcium sulfate in pediatric hematogenous osteomyelitis was satisfactory.

**Supplementary Information:**

The online version contains supplementary material available at 10.1186/s12887-022-03791-4.

## Background

Osteomyelitis is an inflammatory disorder of bone caused by infection leading to necrosis and destruction [1]. The prevalence of osteomyelitis has increased in recent decades with a serious disease burden and socioeconomic impact [2]. Hematogenous osteomyelitis(HO) is a type of osteomyelitis in which bacteria reach bone through hematogenous seeding. The methods for avoiding disease progression are surgical intervention and drainage [3]. Surgical intervention includes decompression, excision of necrotic tissue, sequestrectomy, irrigation and aspiration [4]. Treating the bone loss caused by surgical intervention is a challenge for orthopedic surgeons. Polymethylmethacrylate (PMMA), a nonabsorbable bone filler, was reported to achieve satisfactory curative effects in chronic pediatric osteomyelitis when it was administered in combination with antibiotics into the areas of bone loss caused by surgical intervention [5]. However, there are disadvantages: PMMA is not biodegradable; the material does not allow bone regrowth; and in most cases, an additional surgical procedure is required for removal of the beads and subsequent bone grafting [6–8]. In contrast, calcium sulfate(CS) which can be impregnated with antibiotics, is osteoconductive and does not require a two stage procedure for removal; in addition, CS has been well characterized clinically as a bone void filler [9–12]. Tobramycin, vancomycin and gentamicin are common choices for CS loading [13]. Antibiotic-impregnated CS pellets have been applied in the treatment of pediatric HO and have been reported to yield satisfactory outcomes [14, 15]. As a supplement, the aim of this study was to share our experience with antibiotic-impregnated CS and its efficacy in the treatment of pediatric HO.

## Methods

### Study design and setting

**Fig. 1:**
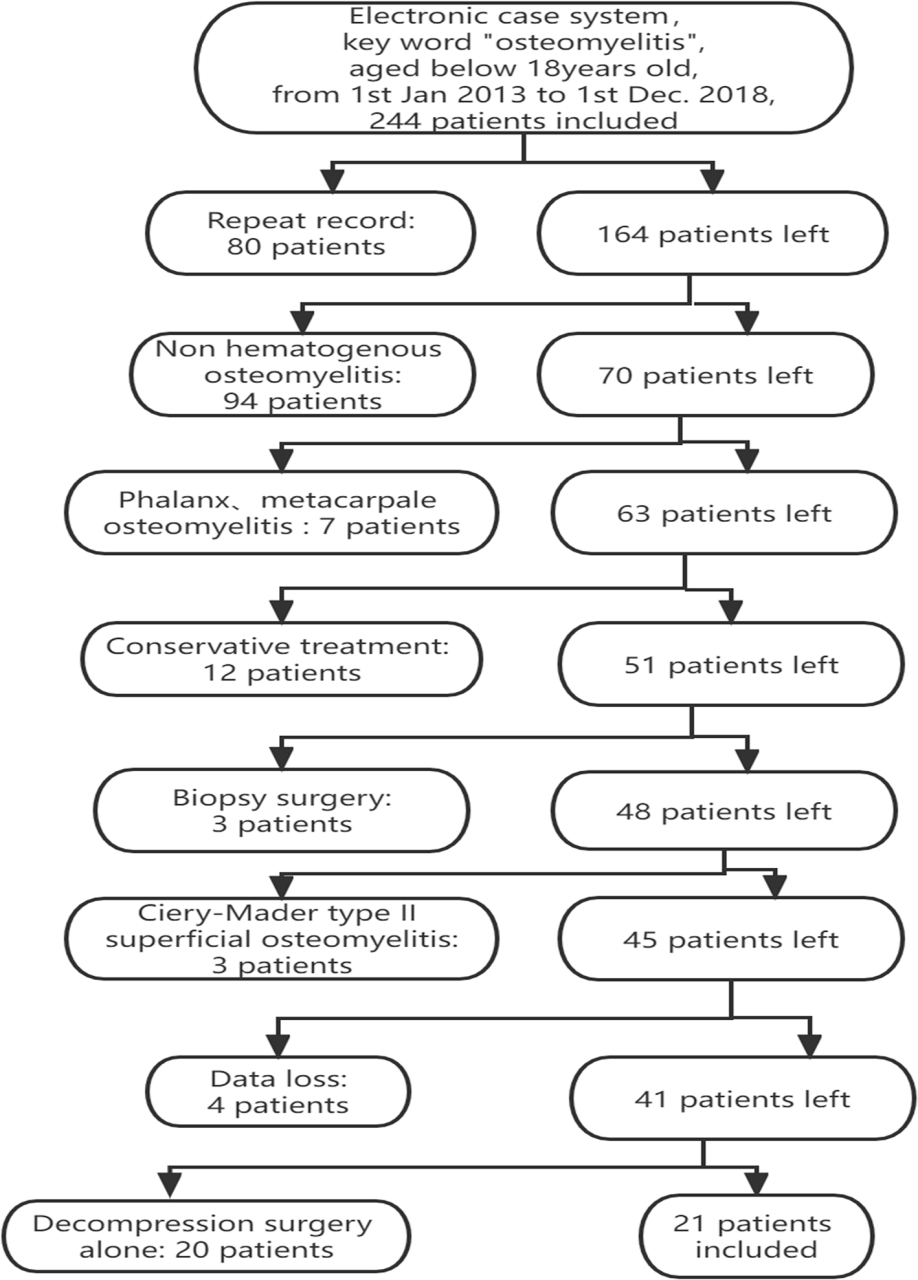
Flow diagram of recruitment for the study

We recruited subjects by searching the electronic medical record system of Southern Medical University Nanfang Hospital using key words (Fig. 1). A case series study of pediatric HO patients treated at our hospital from January 2013 to December 2018 was performed. The main inclusion criteria were as follows: 1) pediatric patients less than 18 years old; 2) patients with HO who underwent surgical intervention and were treated with CS; and 3) patients who remained under follow-up monitoring for at least 24 months. The main exclusion criteria were as follows: 1) patients who suffered from direct spreading osteomyelitis, such as posttraumatic osteomyelitis, or infection after internal fixation; 2) patients who underwent soft tissue surgery and 3) patients who were lost to follow-up. The diagnosis of osteomyelitis was made according to Peltola and Vahvanen's definition [16] or a positive culture. The collected clinical data included demographics, clinical history, serum inflammatory indexes (white blood cell count (WBC), erythrocyte sedimentation rate (ESR), and C-reactive protein (CRP) level), etiology, imaging data, osteomyelitis site, operation process and follow-up duration.

### Descriptions of the experiment, treatment and surgery

**Fig. 2:**
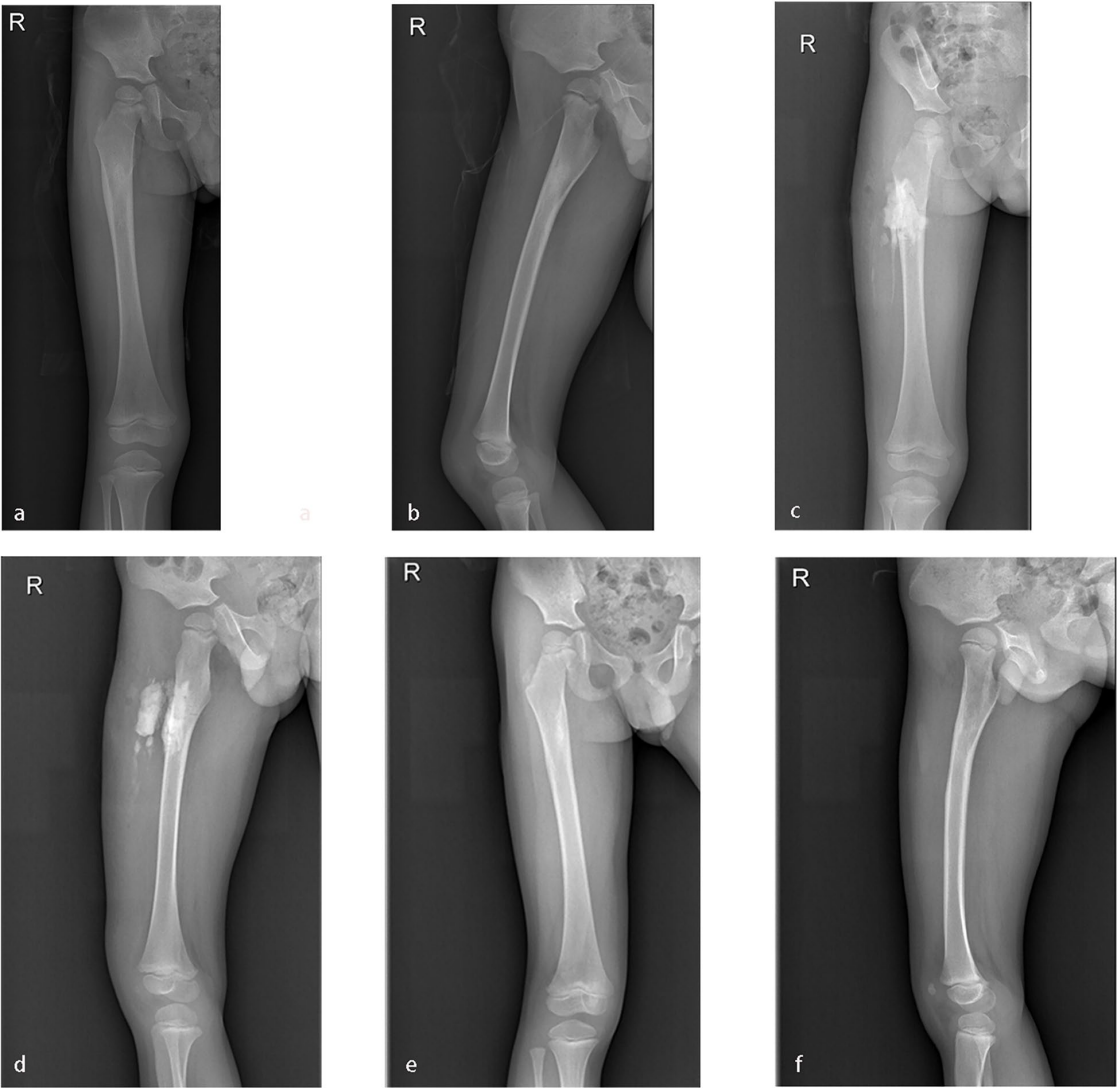
**a**-**b**: A three-year-old boy suffered right proximal femoral HO. **c**-**d**: Vancomycin impregnated CS was used to fill the void left by decompression and debridement. **e**–**f**: X-ray examination indicated good recovery 12 months postoperatively

The surgical approach was guided by preoperative imaging and employed the safest, most direct route to debride all foci of infection. Deep soft-tissue fluid collections, abscesses and subperiosteal tissues were removed. In patients with long bone osteomyelitis, a cortical window was created at the epicenter of infection to enable access to the region. Intramedullary abscesses were debrided with a suitable curet, and the adjacent physis was carefully protected. In patients with calcaneus osteomyelitis, eggshell-like decompression technology [17] was used. The inside abscess was debrided with a suitable curet, and the adjacent physis was carefully protected. The lesions and/or abscesses were sent for biopsy and bacterial culture. Then antibiotic-impregnated CS was applied to fill the void and deliver local antibiotics. One gram vancomycin (or 160 mg of gentamicin or both) and 10 mL of CS were mixed thoroughly using the solvent provided by the manufacturer until a smooth paste was formed (approximately 30 s). The paste was allowed to cure undisturbed for at least 15 min after mixing. The volume of CS varied according to the size of the bone defect. The CS formulation (beads or block-shaped or both) was then placed into the defect as well as the superior-inferior medullary cavity (Fig. 2). The periosteum, subcutaneous tissue and skin were sutured in turn. A cast or external fixation was applied if the bone was unstable after decompression. Bone transport technology was applied in cases of extensive bone defects.

### Aftercare and Follow-up

All patients were routinely given intravenous antibiotics postoperatively. Cefmetazole 100 mg/kg/day was used in 14 patients. Clindamycin 30 mg/kg/day was used in 2 patients who were allergic to cefmetazole. Vancomycin 40 mg/kg/day was used in 5 patients when the pathogenic bacterium showed resistance to cefmetazole. Intravenous antibiotics were administered for a mean of 8.19 days, followed by the administration of oral antibiotics for approximately two weeks. The patients were released when their symptoms improved. The patients were regularly reviewed by the outpatient department or by telephone interview. Infection recurrence was defined as a worsening clinical symptom, continuously increasing serum inflammatory markers, or development of sinus tract and/or continuous bone tissue destruction on X-ray examination. We considered patients in remission of infection when there was an absence of clinical, laboratory, or radiological signs of infection as evaluated during the last medical visit (in patients with a minimum of two years of follow-up) and in patients in which there was no need for reoperation or administration of an extra course of antibiotic therapy at the same site of infection following the end of therapy.

## Results

In all, thirteen males and eight females were included for assessment, and the average age was 10.14 years (range, 3–18 years). The site of onset was the femur in six patients, the tibia in five patients, the calcaneus in three patients, the fibula in three patients, the humerus in two patients, the radius in one patient, and the clavicle in one patient. The average time of onset was 7.74 months (range, 0.25–36 months). The average WBC count was 8.76*10^9^/L (range, 6.03–12.07 *10^9^/L). The average ESR was 44.14 mm/L (range, 26–70 mm/L). The average CRP was 12.35 mg/L (range, 1.03–35.04 mg/L). Thirteen patients were primary surgical pediatric patients. Eight patients had a surgical history at the site of onset (Tables 1 and 2).

**Table 1:** Demographics and clinical outcome of primary operation patients

Patient number	Age/Sex	Bone	Side	Time of onset (months)	History	Symptoms	WBC (10^3^/μl)	ESR (mm/h)	CRP (mg/l)	Etiology/Type of specimen	Comorbidities	Local Antibiotic	Systemic antibiotic/time	Follow up duration (months)	Outcome of treatment
1	16/M	Femur	L	6	Topical application of traditional chinese medicine	Pain, Swelling, Redness	15.35	40	51.8	Negative Intraoperative specimen	-	Vancomycin	Cefmetazole/21 days	55	Cured, Leakage of the incision
2	10/M	Calcaneus	R	12	-	Pain	7.25	17	2.44	Negative Intraoperative specimen	-	Vancomycin	Clindamycin/5 days	31	Cured
3	12/M	Tibia	L	0.25	-	Pain, Swelling,Redness, Fever	11.35	91	190	Negative Intraoperative and blood specimen	-	Vancomycin	Cefmetazole/10 days	54	Cured
4	13/M	Femur	L	2	Arthrocentesis Culture of articular puncture fluid: Staphylococcus aureus	Pain	6.63	52	79.93	S.S.S.S.*aureus* Intraoperative specimen	Suppurative arthritis of the hip	Vancomycin	Cefmetazole/ 17 days	35	Cured
5	3/M	Femur	R	8	-	Limping	13.49	20	37.5	*S. aureus* Intraoperative specimen	-	Vancomycin	Clindamycin/3 days	88	Cured
6	13/F	Femur	R	12	-	Pain, Fever	8.04	40	18.66	*S. aureus* Intraoperative specimen	-	Vancomycin	Cefmetazole/4 days	35	Cured
7	5/F	Tibia	R	1	Topical application of traditional chinese medicine	Pain, Sinus	14.36	70	18.5	*S. aureus* Intraoperative specimen	-	Gentamycin	Cefmetazole/9 days	91	Cured
8	15/M	Clavicle	R	8	-	Pain, Swelling	7.54	6	1.05	*MRSA* Intraoperative and pus puncture specimen	-	Vancomycin	Vancomycin/2 days	43	Cured
9	18/M	Humerus	L	36	-	Pain	8.37	5	1.65	Negative Intraoperative specimen	-	Gentamycin	Cefmetazole/4 days	42	Cured
10	10/M	Radius	L	6	-	Pain	7.31	3	0	Negative Intraoperative specimen	-	Vancomycin	Cefmetazole/6 days	45	Cured
11	12/F	Fibula	L	12	Topical application of traditional chinese medicine	Pain, Sinus	5.99	5	0.11	*MRSA* Intraoperative and sinus specimen	-	Vancomycin	Vancomycin/5 days	69	Cured
12	10/F	Fibula	L	0.33	Blood culture: MRSA	Pain, Fever	21.78	102	48.77	*MRSA* Intraoperative and blood specimen	Acute tonsillitis, Septicemia	Vancomycin	Vancomycin/14 days	59	Cured, Osteolysis
13	10/M	Tibia	R	1	-	Pain	7.54	23	1.78	Negative Intraoperative specimen	G6PD Deficiency	Vancomycin	Cefmetazole/7says	35	Cured

*Abbreviation: WBC* White Blood Cell count, *ESR* Erythrocyte Sedimentation Rate, *CRP* C-Reactive Protein, *S aureus* Staphylococcus aureus, *MRSA* Methicillin-resistant S.aureus, *G6PD* Glucose-6-phosphate Dehydrogenase

**Table 2:** Demographics and clinical outcome of reoperation patients

Patient number	Age/Sex	Bone	Side	Time of onset (months)	History	Symptoms	WBC (10^9^/l)	ESR (mm/h)	CRP (mg/l)	Etiology/Type of specimen	Comorbidities	Local Antibiotic	Systemic antibiotic/time	Follow up duration (months)	Outcome of treatment
1	6/F	Fibula	L	5	Decompression surgery 5 months prior	Pain, Fever,	8.01	44	3.66	*S. aureus* Intraoperative specimen	-	Vancomycin + Gentamycin	Cefmetazole/6 days	31	Cured
2	14/M	Humerus	R	6	Decompression surgery 6 months prior	Pain,Sinus	13.64	92	87.18	*S. aureus* Intraoperative specimen	-	Gentamycin	Cefmetazole/8 days	58	Cured
3	15/M	Femur	L	3	Decompression surgery 1 month prior	Pain	6.75	12	4.47	*aureus* Intraoperative specimen	-	Vancomycin	Cefmetazole/8 days	37	Cured
4	3/M	Calcaneus	R	9	Debridement surgery of soft tissue 9 months prior	Pain,Sinus	7.72	38	28.2	Negative Intraoperative specimen	-	Vancomycin	Cefmetazole/3 days	36	Cured
5	6/F	Tibia	L	5	Multiple debridement surgery 4 months prior	Pain,Sinus	8.49	41	4.81	*MRSA* Intraoperative specimen	-	Vancomycin	Vancomycin/7 days	40	Cured, Knee valgus deformity
6	10/M	Calcaneus	L	1	Debridement surgery 7 days prior	Pain,Unclosed wound	5.84	36	3.3	*cloaca* Intraoperative specimen	-	Vancomycin	Cefmetazole/10 days	53	Cured
7	5/F	Femur	R	5	Decompression surgery 5 months ago, pathological fracture 3 months prior	Pain, Swelling, Pathological fracture	7.14	44	8.1	*MRSA* Intraoperative specimen	-	Vancomycin	Vancomycin/12 days	31	Cured, Retardation of bone growth
8	7/F	Tibia	L	24	Decompression surgery 12 months prior	Pain	6.28	14	0.5	Negative Intraoperative specimen	-	Vancomycin	Cefmetazole/11 days	37	Cured, Acceleration of bone growth

*Abbreviation: WBC* White Blood Cell count, *ESR* Erythrocyte Sedimentation Rate, *CRP* C-Reactive Protein, *S. aureus* Staphylococcus aureus, *E. cloacae* Enterobacter cloacae, *MRSA* Methicillin-resistant S.aureus, *G6PD* Glucose-6-phosphate Dehydrogenase

This study included twenty-one patients with HO. The etiology was *Staphylococcus aureus* (*S. aureus*) in seven patients, *Methicillin-resistant S.aureus* (*MRSA*) in five patients, and *Enterobacter cloacae (E. cloacae*) in one patient, and the culture was negative in eight patients. The average follow-up was 47.9 months (range, 31–91 months). The infection recurrence rate was 0%(0/21) and infection eradication was achieved in all of the patients (Tables 1 and 2).

**Fig. 3:**
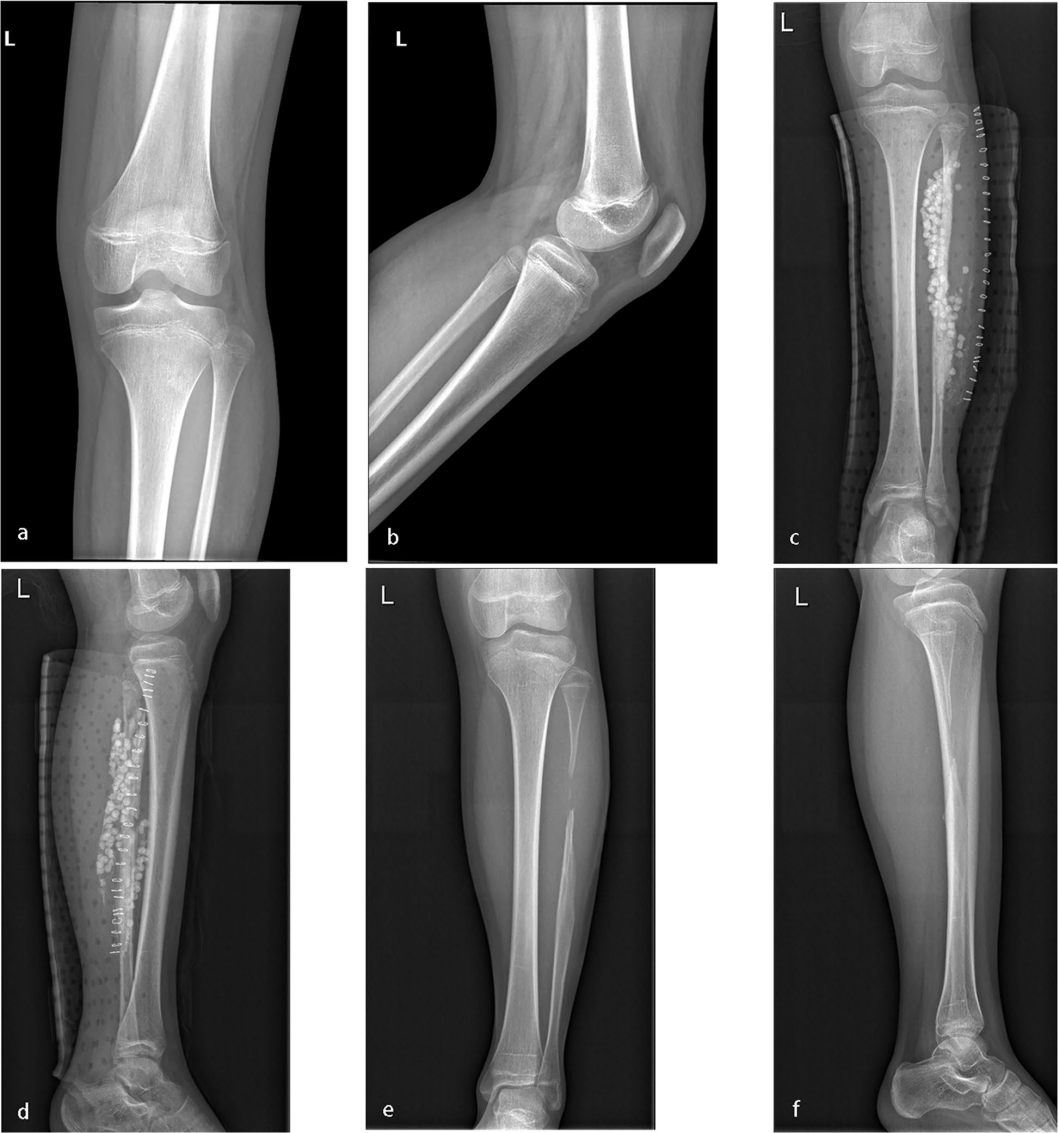
**a**-**b**: A ten-year-old girl suffered left proximal fibular HO. **c**-**d**: Vancomycin impregnated CS was used to fill the void left by decompression and debridement. **e**–**f**: X-ray examination indicated osteolysis 18 months postoperatively

Thirteen patients underwent a primary operation. Among these patients, three had a history of traditional Chinese medicine usage. Of these three patients, two had a clinical manifestation of sinus leakage at the site of onset preoperatively, and one had incision leakage postoperatively (Table 1). The duration of incision leakage was approximately four weeks, at which point the leakage disappeared. One patient with fibular osteomyelitis who underwent the surgical procedures was discovered to have osteolysis at a followed-up visit (Fig. 3). None of the patients had pain or walking or adjacent joint activity limitations. All of the patients had a daily life comparable with that of their normal peers.

**Fig. 4:**
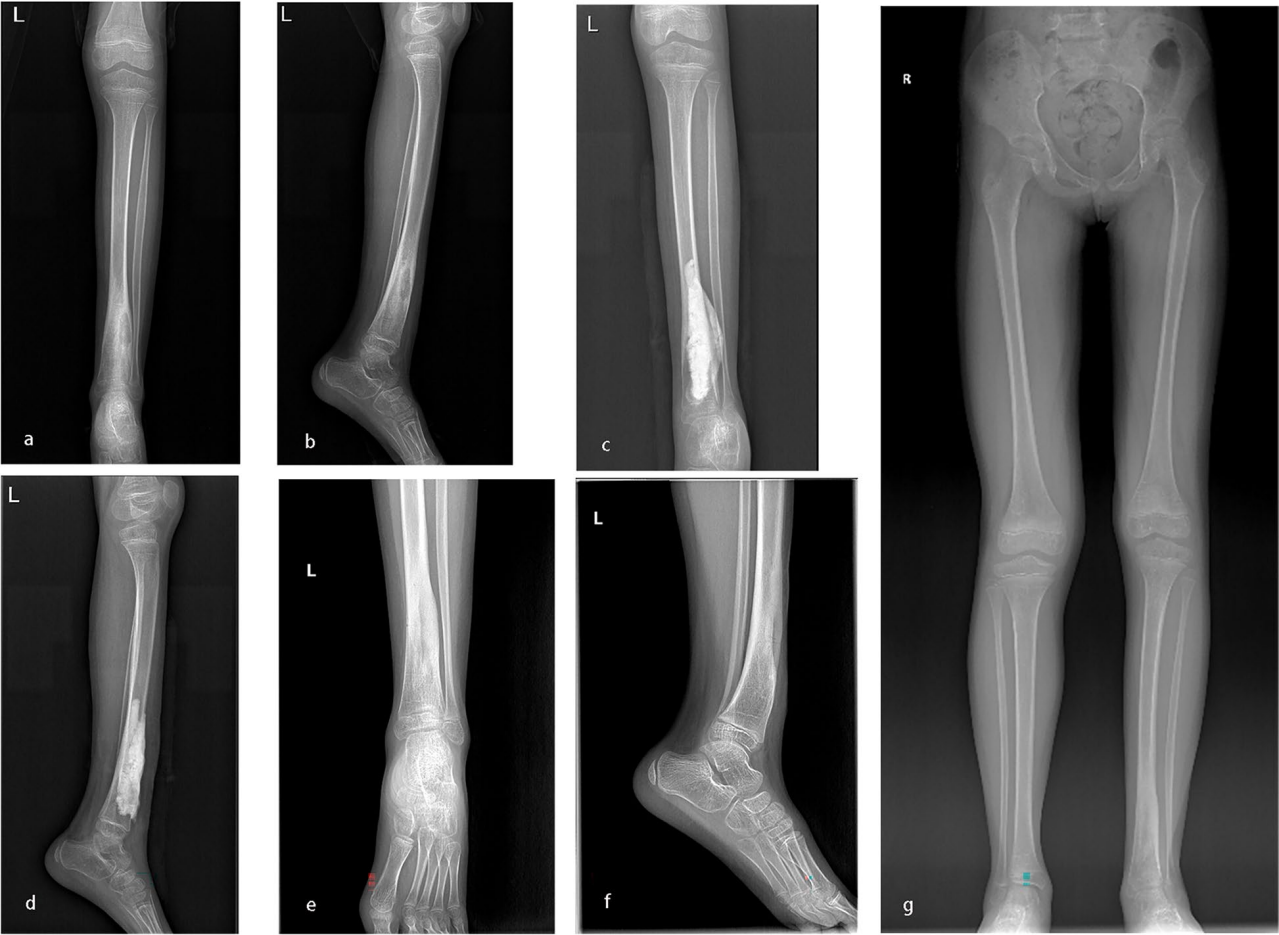
**a**-**b**: A seven-year-old girl suffered left distal tibial HO. **c**-**d**: Vancomycin impregnated CS was used to fill the void left by decompression and debridement. **e**–**g**: X-ray examination indicated acceleration of bone growth 12 months postoperatively

**Fig. 5:**
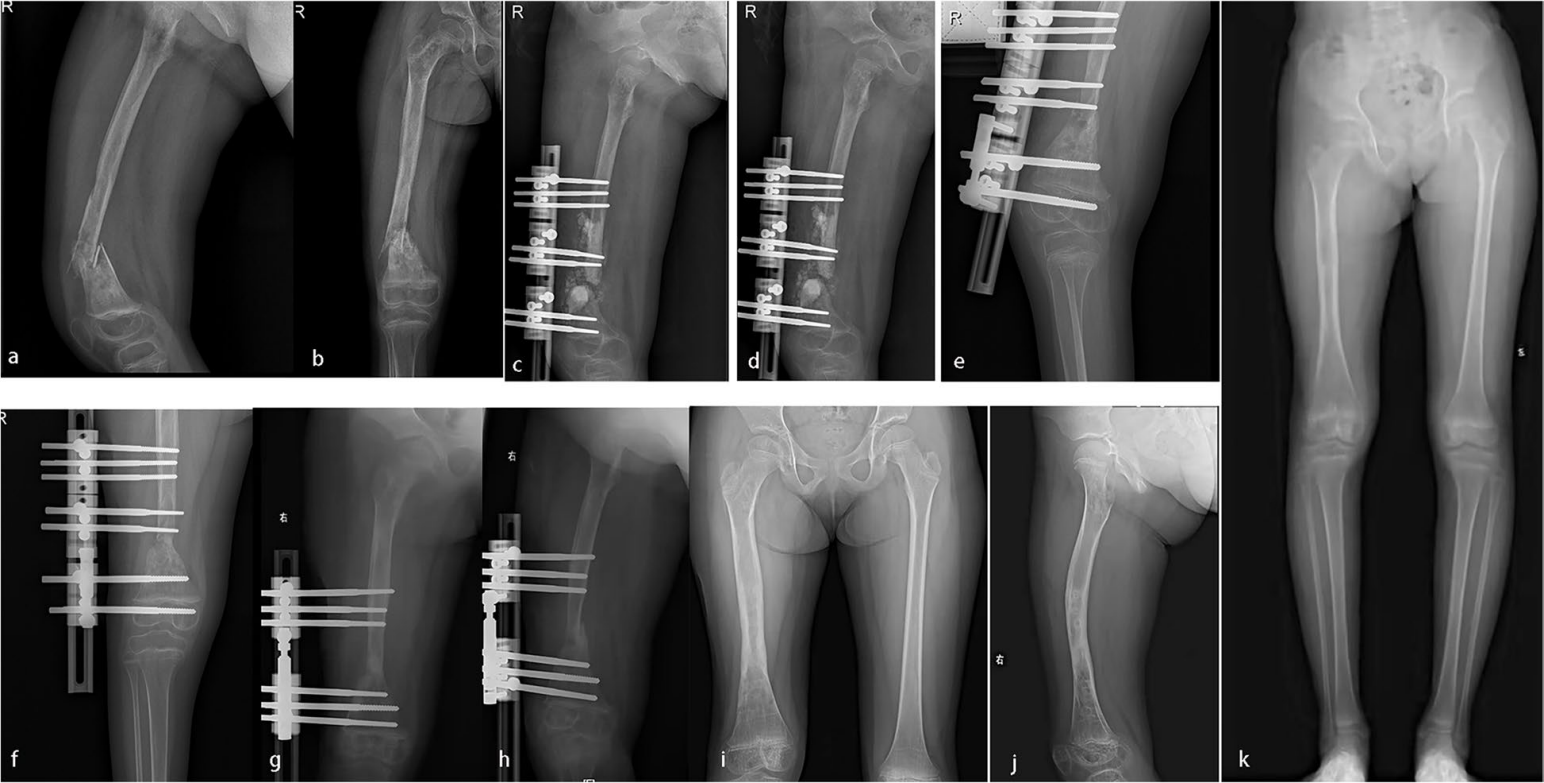
**a**-**b**: A five-year-old girl suffered right distal femoral HO and pathological fracture. **c**-**d**: Vancomycin impregnated CS was used to fill the void left by decompression and debridement, and the bone transport technique was applied. **e**–**f**: Bone transport was finished 3 months postoperatively. **g**-**h**: Removing of the distal screw and adjusting the external fixation device. **i**-**k**: X-ray examination indicated retardation of bone growth and lower limb discrepancy 19 months postoperatively

**Fig. 6:**
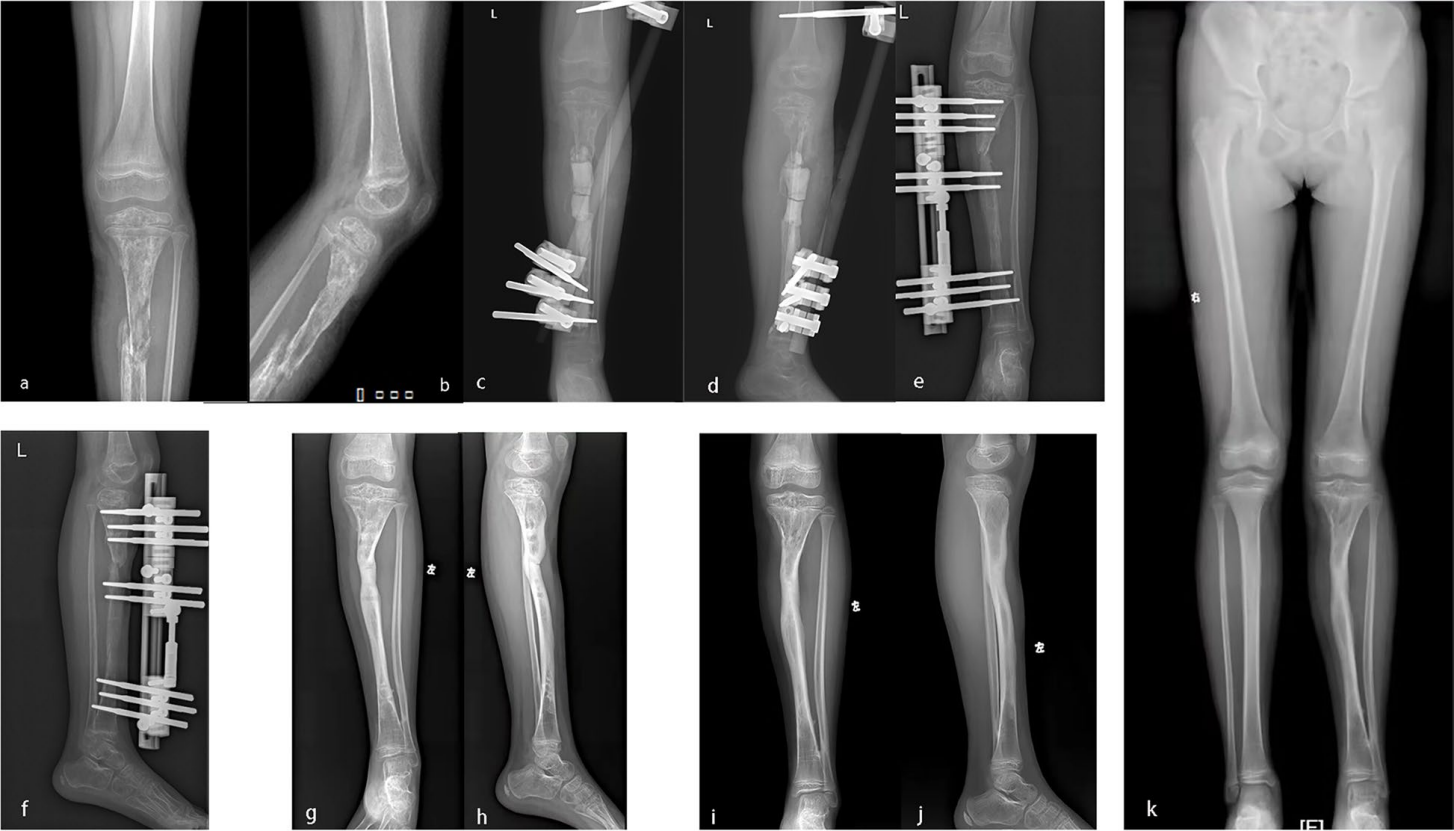
**a**-**b**: A six-year-old girl had suffered left proximal tibia HO. **c**-**d**: Vancomycin impregnated CS was placed in the void spaces left by decompression and debridement. The tibia was fixed by a temporary external fixator. **e**–**f**: A terminal unilateral external fixator was used for bone transport. Bone transport was finished 9 months postoperatively. **g**-**h**: External fixation was removed 18 months postoperatively. **i**-**k**: The latest X-ray was taken at 34 months postoperatively. The girl showed genu valgus deformity

Eight patients had a history of decompression or debridement at the site of onset. Among these patients, five had a serious clinical manifestation: three had sinus leakage, one had an unclosed wound and one had a pathological fracture (Table 2). Moreover, among these patients, three had serious complications: one with tibial osteomyelitis who underwent the surgical procedures experienced acceleration of bone growth as a complication (Fig. 4), one with femoral osteomyelitis who underwent the surgical procedures and bone transport experienced retardation of bone growth (Fig. 5), and one with tibial osteomyelitis who underwent the surgical procedures and bone transport developed genu valgus deformity (Fig. 6). These three patients needed to walk with a crutch to avoid bear load in the first month removing the external fixation. Their myodynamia and motion of adjacent joints were slightly limited in the initial three months after removing the external fixation. After six months removing the external fixation, they had no pain and no walking limitations with appropriate treatment, such as heightened insoles and knee braces. The remaining patients had no pain nor walking nor adjacent joint activity limitations and had a daily life comparable to that of their normal peers.

## Discussion

HO is a type of pediatric musculoskeletal infection. The strategies for avoiding disease progression are surgical intervention and drainage [3]. HO is the most difficult condition to understand in the realm of pediatric musculoskeletal infection and continues to present a significant clinical challenge [18]. Copley et al. [4] presented a surgical approach consisting of bone decompression and curettage of intramedullary abscesses in 2009. However, they did not describe filling of the void left by surgery. Bar-On et al. [5] reported the successful treatment of four patients with chronic pediatric osteomyelitis with intramedullary reaming and antibiotic-impregnated PMMA in 2010. This was the first report of a nonabsorbable local antibiotic release system applied in pediatric osteomyelitis. Antonio Andreacchio et al. [14] reported the successful treatment of twelve pediatric patients with chronic osteomyelitis by debridement and filling with antibiotic-impregnated CS in 2019. This was the first report of an absorbable local antibiotic release system applied in pediatric osteomyelitis. Vikas Ellur et al. [15] reported the successful treatment of thirty-four pediatric patients with chronic osteomyelitis by debridement and filling with antibiotic-impregnated CS in 2021. In our study, we report the successful treatment of twenty-one pediatric patients with HO by decompression, debridement and filling with antibiotic-impregnated CS. All of the patients were infected via the hematologic route. Our study is a supplement to these previous studies and presents a deeper understanding of the application of CS in HO.

Masquelet [19] proposed a two-step operation technique for the treatment of chronic osteomyelitis. The first step is sharp debridement of necrotic tissue, abundant lavage, sequestrectomy, and removal of any hardware and/or foreign bodies, followed by delivery of antibiotic-impregnated PMMA. The second step is an additional surgical procedure required for removal of the beads and subsequent bone grafting. This technique can be used in cases of initial infection and to control established infection before final bone reconstruction. Although the application of antibiotic-impregnated PMMA has shown an excellent curative effect in the eradication of infection, its deficiencies have been obvious. First, PMMA has the potential to serve as a reservoir for recurrent infection if left in the void past the period of effective antibiotic elution due to its nonbiodegradable nature [20, 21]. Second, PMMA does not contribute to bone regrowth and requires an additional surgical procedure to be removed. This second procedure delays the healing process and increases the cost [22]. Additionally, other disadvantages include the increased risk of the development of antibiotic-resistant organisms and decreased immune function of the host with PMMA application [20, 23, 24]. Moreover, donor site complications such as instability, infection and fatigue fractures occur when autogenous iliac bone grafting is applied [22]. Therefore, CS, as an absorbable material, has drawn clinical attention. Antibiotic-impregnated CS has unique advantages. First, it exhibits characteristic steady and gradual resorption, it is osteoconductive, and it does not require an additional surgical procedure to be removed [25–27]. Second, antibiotic-impregnated CS has been found to be osteoinductive, suggesting that it can induce the differentiation of bone marrow mesenchymal stem cells into osteoblasts [28]. Additionally, antibiotic-impregnated CS has been found to induce the formation of a vascularized membrane that can prevent graft resorption and create a favorable microenvironment for vascularization and corticalization, thus promoting bone formation [29]. In our study, the infection was eradicated in all 21 patients and none of them required additional surgery. These results confirmed the curative effect of antibiotic-impregnated CS in eradicating the infection.

Although any water-soluble antibiotic can be incorporated into CS, the ideal antibiotic remains controversial. Vancomycin, gentamicin and tobramycin were the common choices for CS loading. In a vitro experiment, vancomycin and tobramycin impregnated materials had similar germicidal properties and elution efficiency [11]. Furthermore, a systematic review indicated that the choice of tobramycin-loaded CS or vancomycin combined with gentamicin-loaded CS did not affect the eradication rate and the incidence of postoperative complications in chronic osteomyelitis patients [13]. The choice of a local antibiotic depends on the local epidemiological data in patients who have no accurate bacterial data. We chose vancomycin empirically for gram-positive patients or patients who were highly suspected to have *S.aureus* infection and gentamicin for gram-negative patients. We chose vancomycin and gentamicin together empirically for patients with hard-to-identify bacterial infections. Compared with long-term intravenous antibiotics, local antibiotics have unique advantages. One advantage is the higher and more effective concentrations that can be obtained in the local area of infected bone through local antibiotic applications over a prolonged period of time. Another is the prevention of adverse events related to systemic chemotherapy and the reduced risk of systemic toxicity [30]. Zhang et al. [31] measured the blood vancomycin levels of 24 osteomyelitis patients locally treated with vancomycin-impregnated CS beads (a dose ranging from 1.5 ml to 5 ml at a ratio of 1 g of vancomycin: 5 ml of calcium sulfate). The results showed that the mean blood vancomycin level was still within a safe range for application. P. Wahl et al. [32] found that when 6 g of vancomycin was applied locally, the systemic concentration remained within a safe range, and the local concentration was still below the reported cellular toxicity thresholds. In our study, intravenous and oral antibiotics were administered to patients over the short term. Adverse events related to systemic chemotherapy and systemic toxicity did not occur.

Ping-chung Leung et al. [33] reported that the topical application of traditional Chinese medicine presented satisfactory results in plantar fasciitis, nonundisplaced metatarsal fracture, Tennis elbow and de-Quervain's disease. The authors indicated that pain relief and inflammation control were the advantages of topical traditional Chinese medicine administration. However, the authors did not describe the condition of the skin. In our study, three patients with no history of surgery were treated with topical traditional Chinese medicine by their parents. Among these patients, two developed sinus leakage preoperatively and one of them had incision leakage postoperatively. Aseptic exudation is one of the disadvantages of CS [30, 34]. A commonly reported observation associated with the surgical use of CS is fluid discharge from the wound/surgical site, occurring in 4% to 51% of patients [11, 22, 35]. The reported duration of fluid discharge is variable, ranging from 2 to 24 weeks [35]. In our study, the duration of fluid discharge was approximately four weeks. We hypothesize that the application of topical traditional Chinese medicine may damage the skin and increase the risk of sinus and aseptic exudation caused by CS. How to prevent aseptic exudation after antibiotic-impregnated CS use is an urgent problem to be solved. In view of this, we present some advice from our own experience: 1. prevent the CS from becoming too wet; 2. place the CS in an area rich with soft tissue; and 3. do not use topical traditional Chinese medicine.

Osteolysis is the most frequent complication of total joint arthroplasty and internal fixation [36, 37]. The mechanisms underlying osteolysis mainly include the following: 1. inflammation caused by inflammatory stimuli; 2. inflammation caused by stimulation of innate immune receptors; 3. regulation of debris-induced inflammation; and 4. inflammation-associated bone resorption [37]. The treatment of osteolysis consists of bone grafting using either bone allografts or bone-graft substitutes (such as various osteoconductive or osteoinductive materials) or combinations thereof [38]. CS itself was developed as a bone-graft substitute that can cure osteolysis. However, in our study, 1 patient treated with CS developed osteolysis (Fig. 3). We speculate that there may be two reasons for this outcome: 1. stimulation caused by bone decompression and debridement; and 2. stimulation caused by the vancomycin-impregnated CS.

The disease of patients who had a history of decompression or debridement at the site of onset was more serious than that of patients who had no history of surgery. In our study, more serious clinical manifestations, such as sinus leakage, unclosed wounds, and pathological fractures, occurred in 62.5% (5/8) of these patients. We do not know how decompression or debridement surgery proceeded in these patients at other hospitals. However, we know that antibiotic-impregnated CS was not applied to the void left by decompression or debridement in these patients. We estimated that failure to apply antibiotic-impregnated CS was a contributing factor of the serious clinical manifestations.

Bone growth has been found to be affected by systemic and local hormonal pathways and mechanical loading [39]. In our study, two patients had experienced abnormal bone growth as a serious complication. One patient experienced acceleration of bone growth, and the other experienced retardation of bone growth (Figs. 4 and 5). We believe that the number of surgeries and the stimulation by CS may have resulted in abnormally accelerated or retarded bone growth.

The bone transport technique refers to the production of new bone between vascular bone surfaces created by an osteotomy and separated by gradual distraction [40]. In our study, two patients who had large bone defects left by surgical intervention underwent treatment with this technique (Figs. 5 and 6). These two patients had a history of decompression or debridement at the site of onset without the application of antibiotic-impregnated CS. As a result, one of them developed genu valgus deformity as a complication. Fortunately, these patients had no pain and no limitations in walking or squatting.

## Limitations

This study had a number of limitations. First, this study was not a retrospective control study, and comparisons were not performed to confirm the results. Second, this study was a small-sample retrospective study, and only twenty-one patients were included. However, the 100% infection eradication rate and the rarity of complications are information that can supplement the findings of existing studies. We expect large-sample randomized controlled trials to confirm our findings.

## Conclusions

Although noninfectious complications occurred, such as incision leakage, osteolysis, and acceleration or retardation of bone growth, the curative effect of antibiotic-impregnated CS in pediatricHO was satisfactory.

## Supplementary Information

**Additional file 1.**

## Data Availability

The data supporting our findings come from Southern Medical University Nanfang Hospital. The datasets generated during and/or analyzed during the current study are available from the corresponding author on reasonable request.
