# Supplementary material for: Antibiotic-impregnated calcium sulfate for the treatment of pediatric hematogenous osteomyelitis
Source: BMC Pediatr. 2022 Dec 23;22:732. doi: 10.1186/s12887-022-03791-4 (PMC9783740; doi:10.1186/s12887-022-03791-4)
Supplement: Supplementary file 1 — Additional file 1. [file 12887_2022_3791_MOESM1_ESM.pdf]

Primary surgery

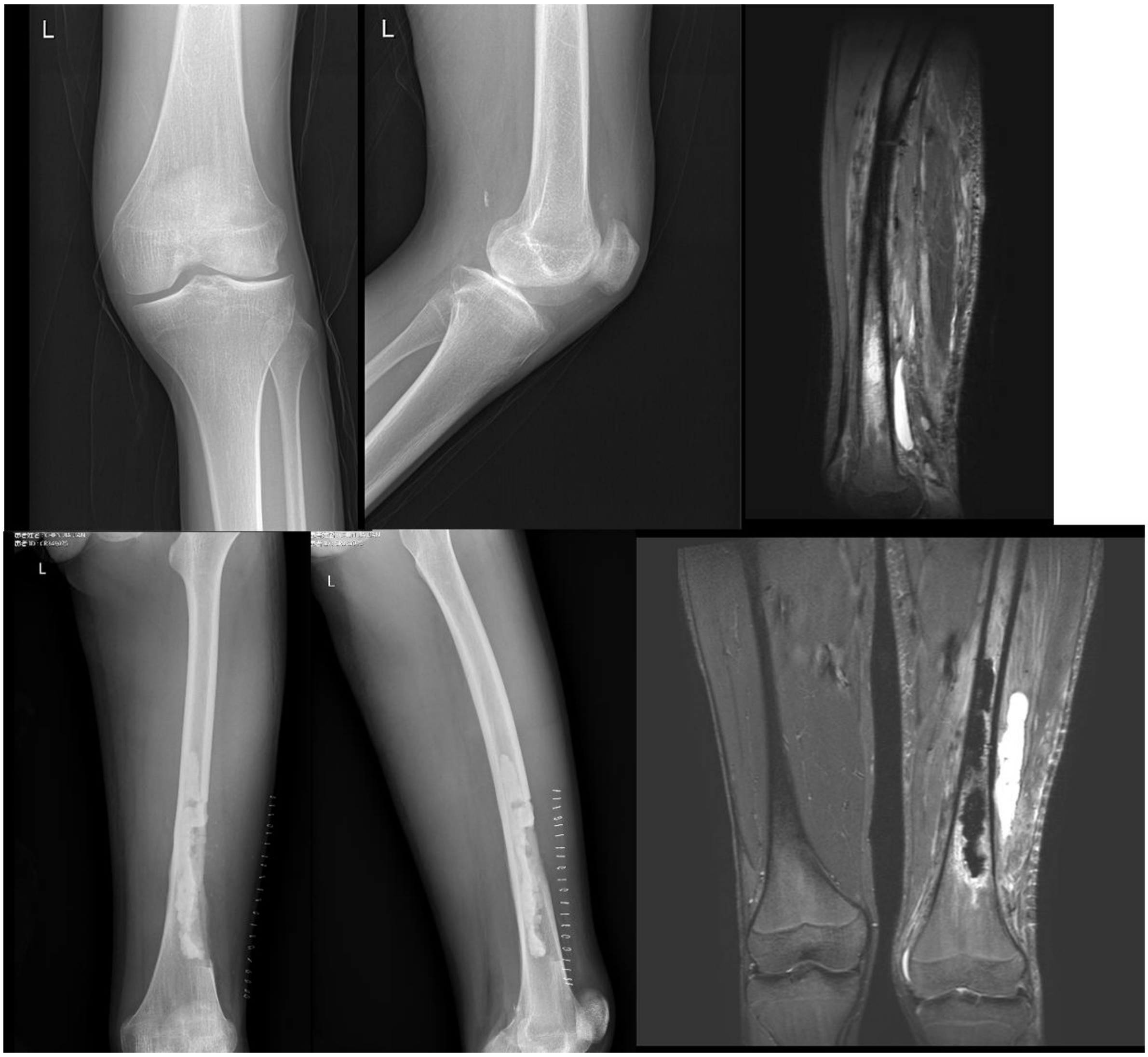

CASE 1. A 16 years old boy with a femur osteomyelitis: preoperative X-ray and MRI, postoperative X-ray.

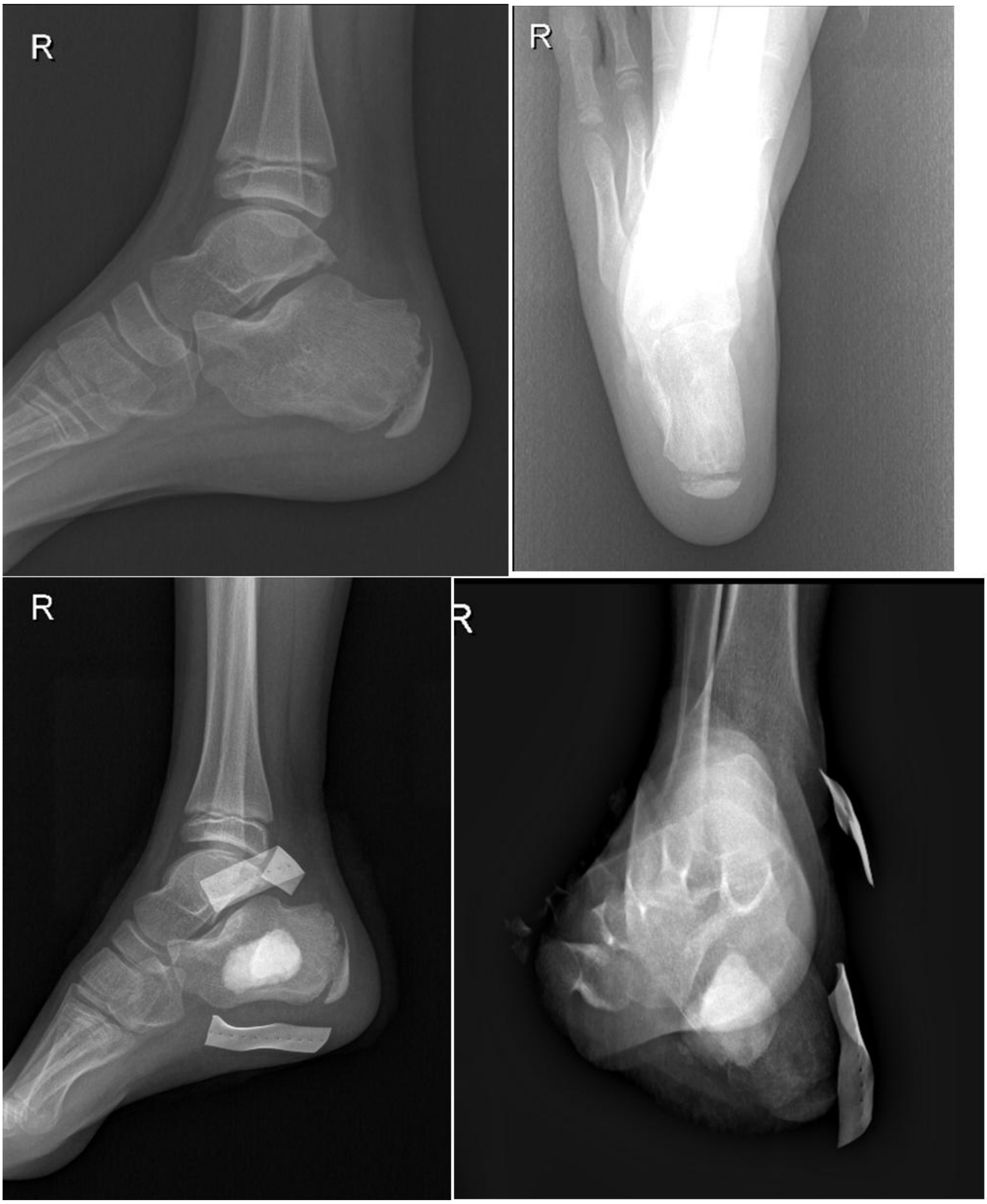

CASE 2. A 10 years old boy with a calcaneus osteomyelitis: preoperative X-ray, postoperative X-ray.

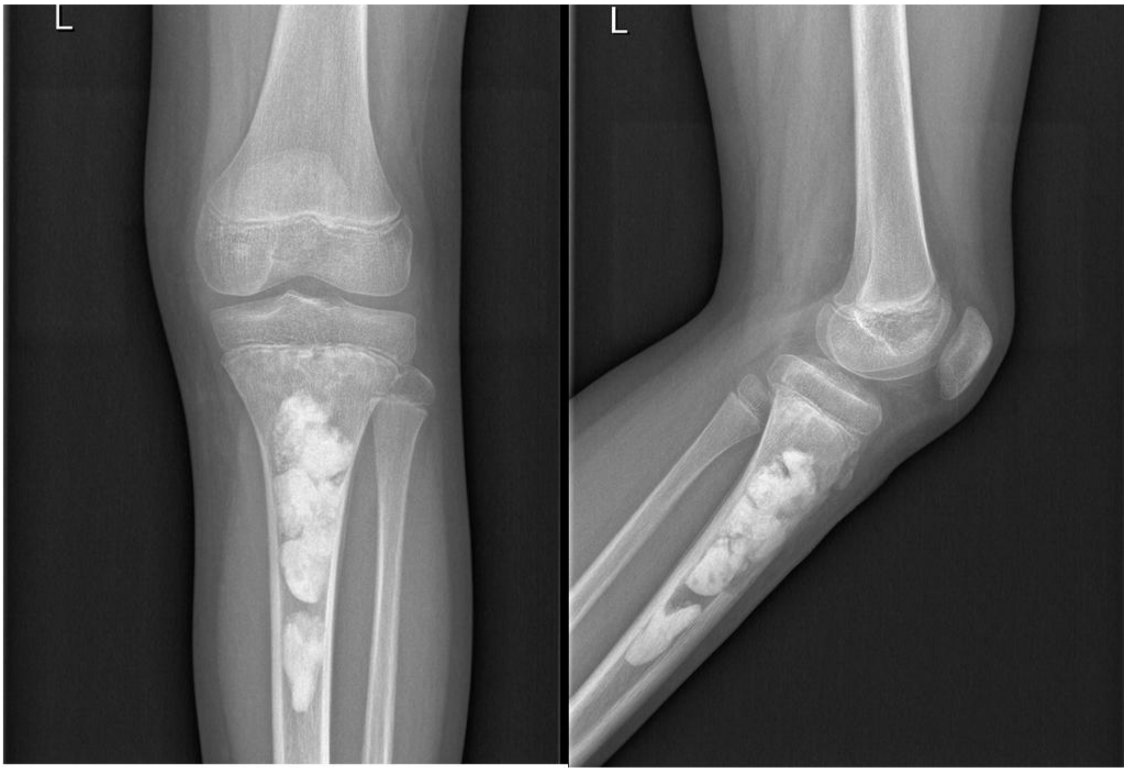

CASE 3. A 12 years old boy with a tibia osteomyelitis: postoperative X-ray.

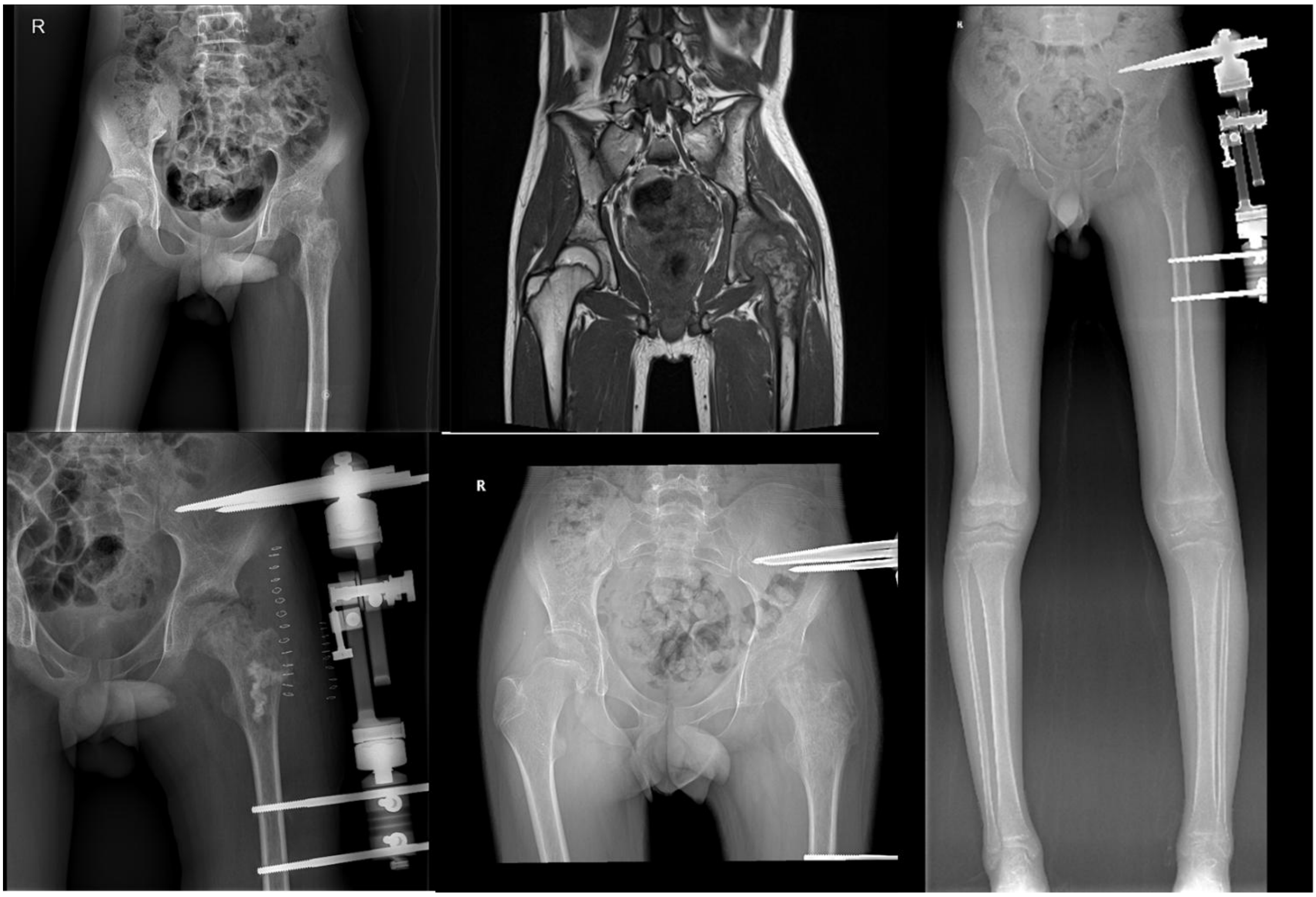

CASE 4. A 13 years old boy with a femur osteomyelitis: preoperative X-ray and MRI, postoperative X-ray. An external fixation was used for stable.

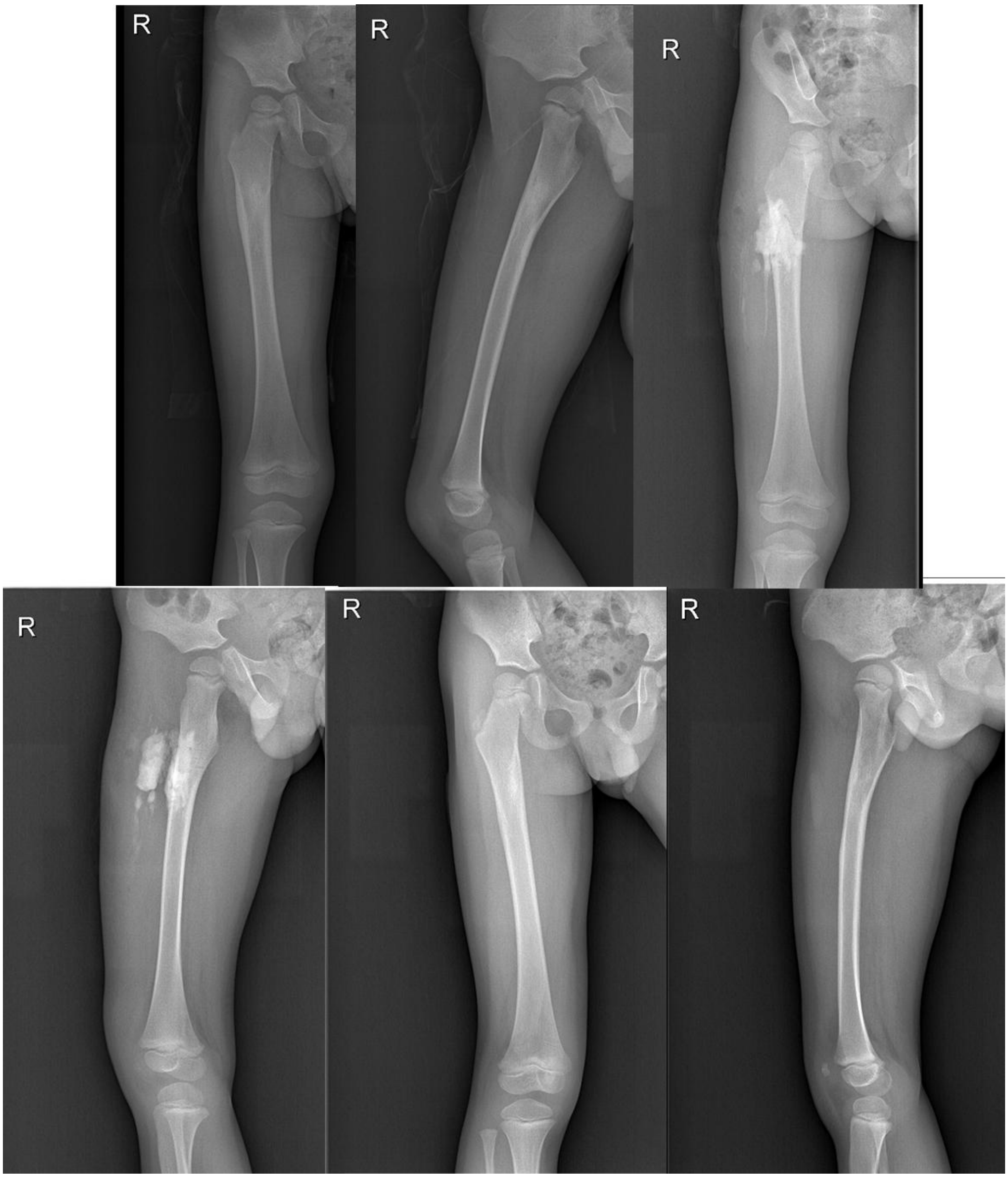

CASE 5. A 3 years old boy with a femur osteomyelitis: preoperative X-ray, postoperative X-ray.

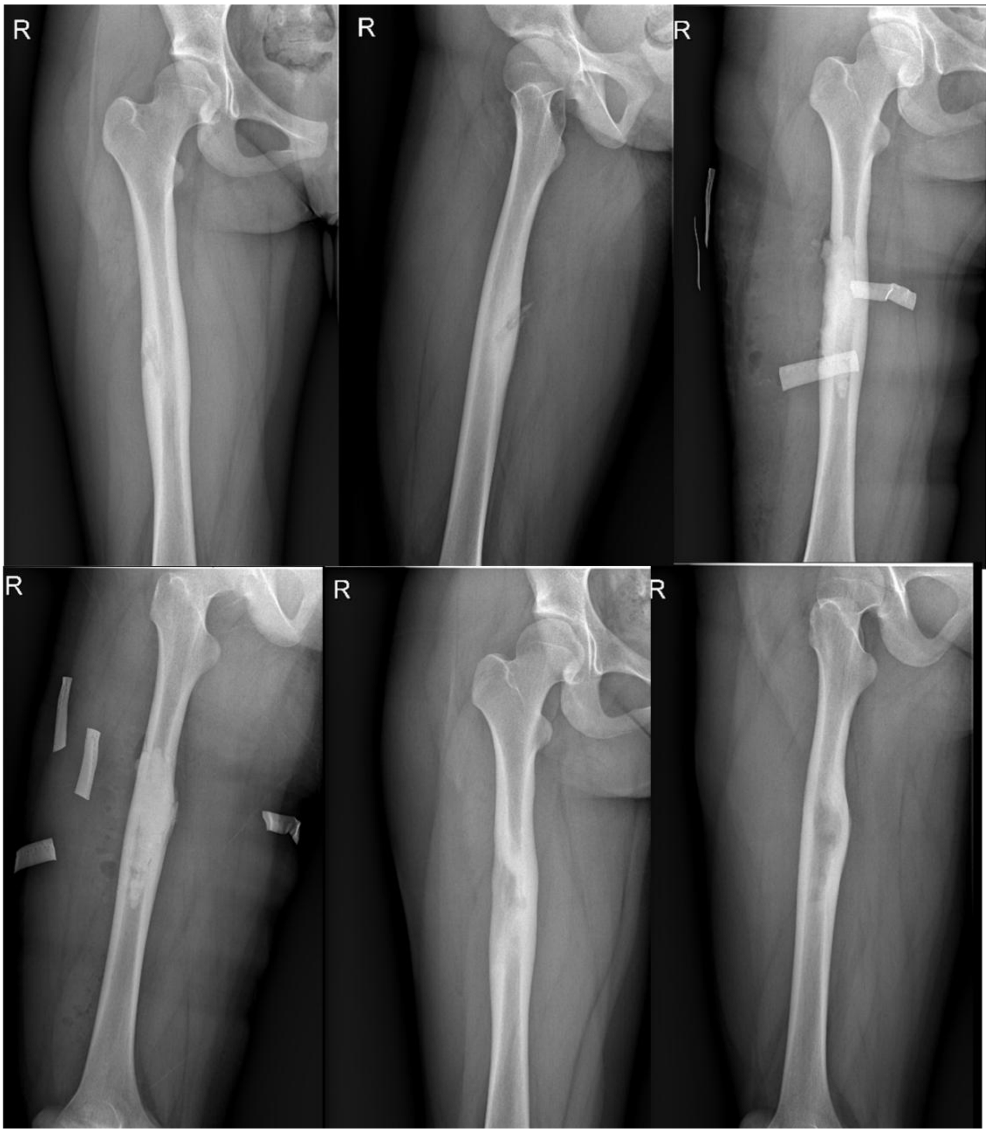

CASE 6. A 13 years old girl with a femur osteomyelitis: preoperative X-ray, postoperative X-ray.

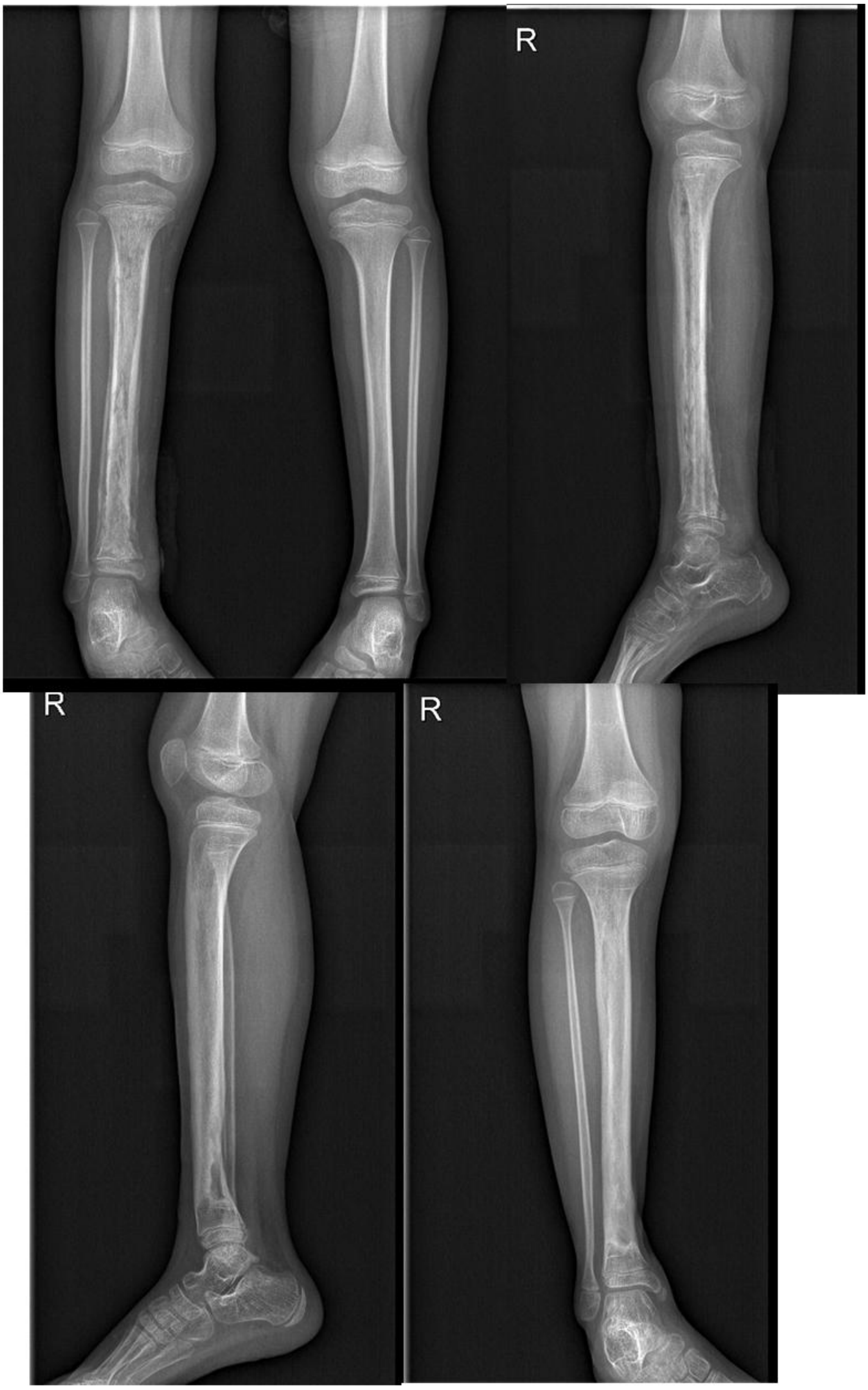

CASE 7. A 5 years old girl with a tibia osteomyelitis: preoperative X-ray, postoperative X-ray.

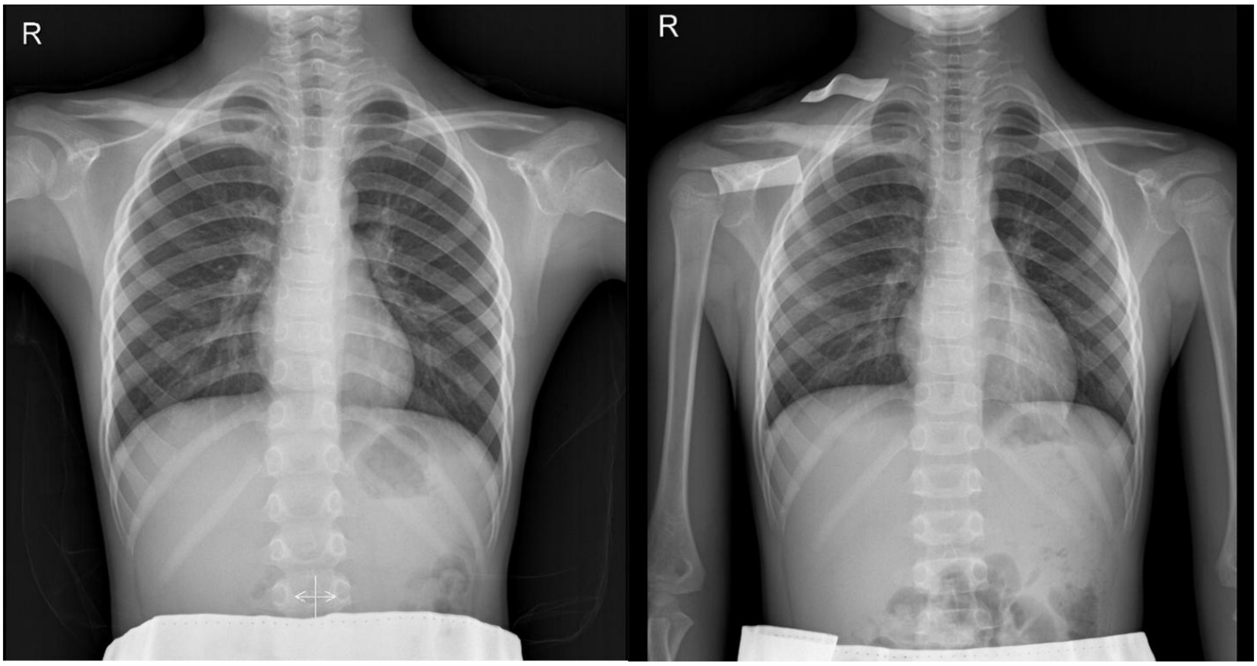

CASE 8. A 15 years old boy with a clavicle osteomyelitis: preoperative X-ray, postoperative X-ray.

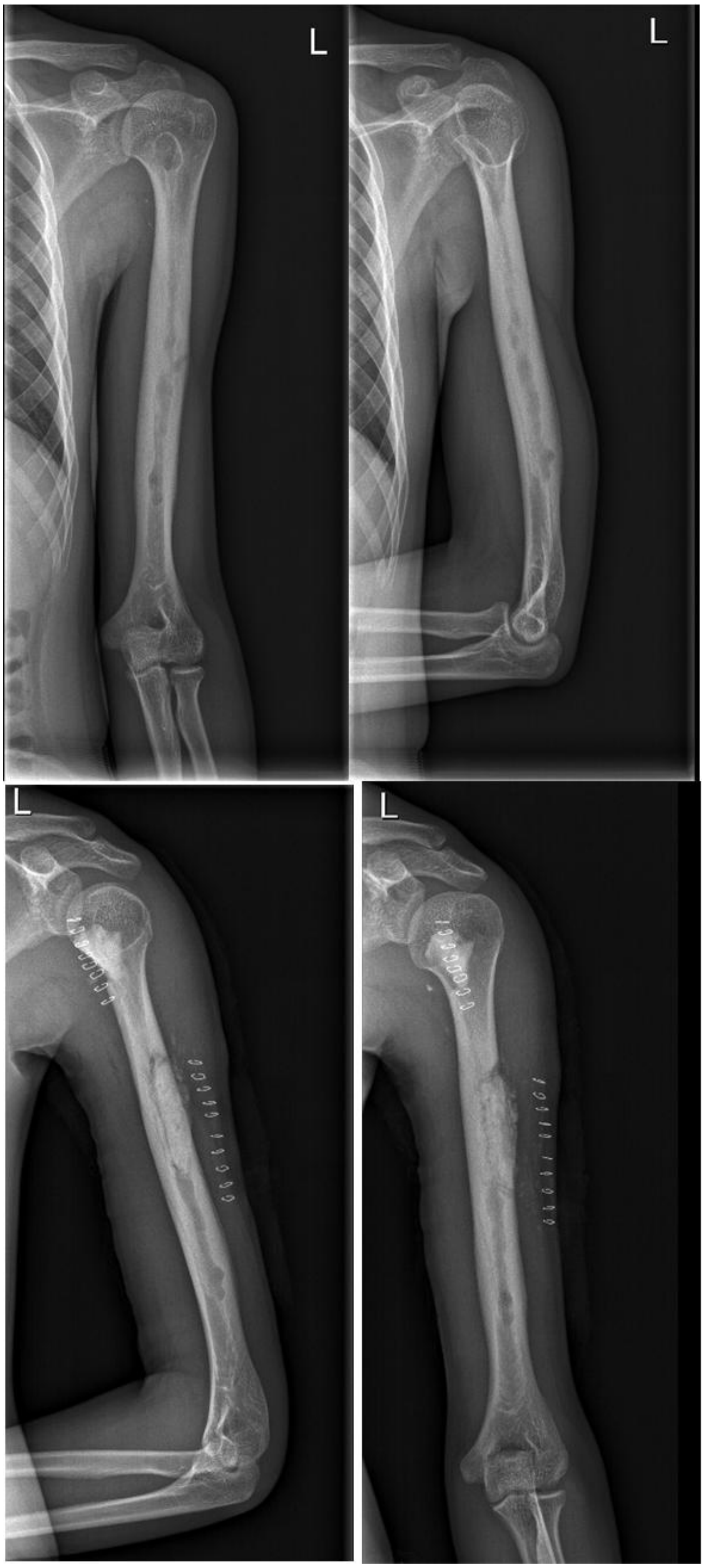

CASE 9. A 18 years old boy with a humerus osteomyelitis: preoperative X-ray, postoperative X-ray.

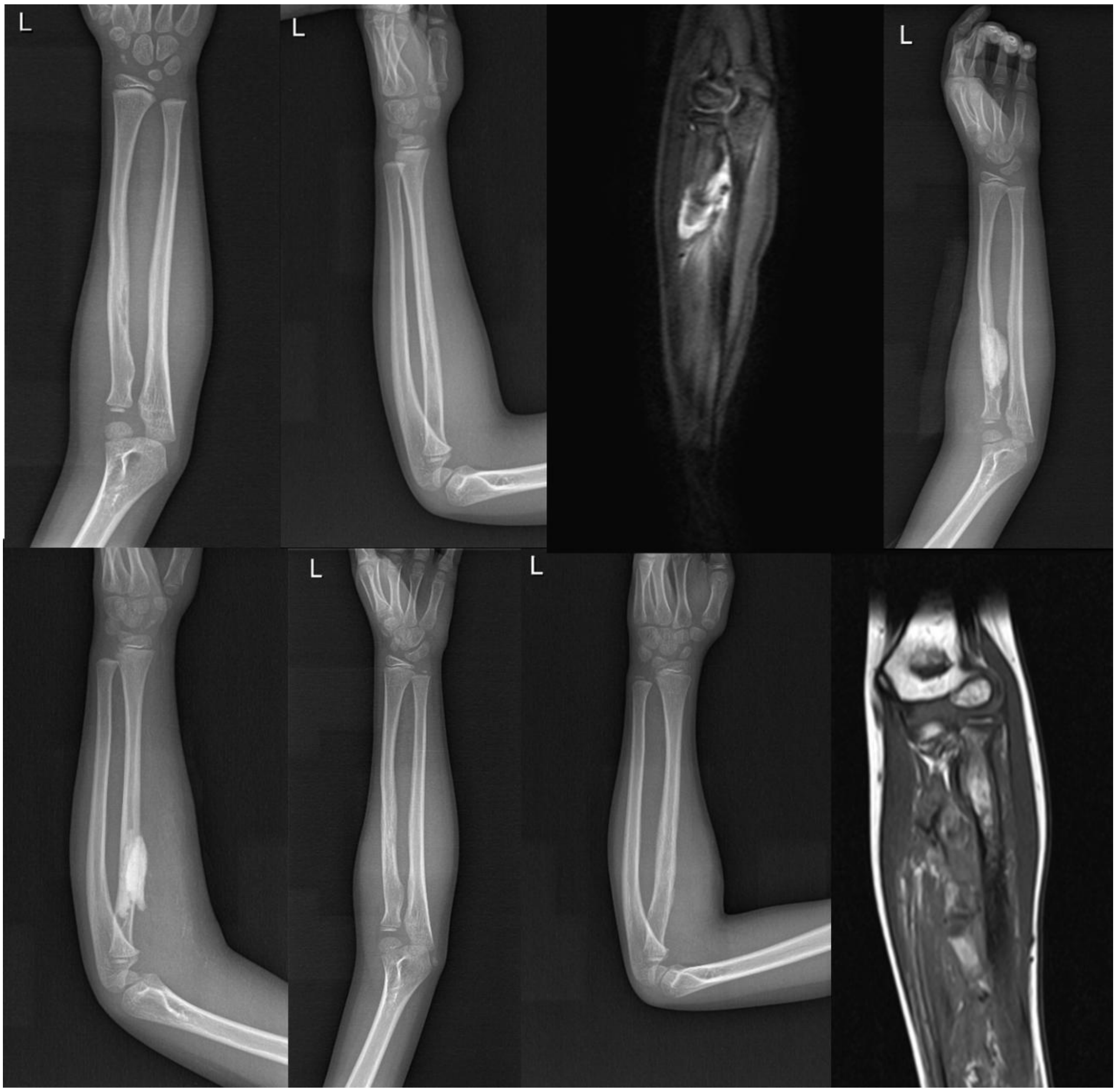

CASE 10. A 10 years old boy with a radius osteomyelitis: preoperative X-ray and MRI, postoperative X-ray.

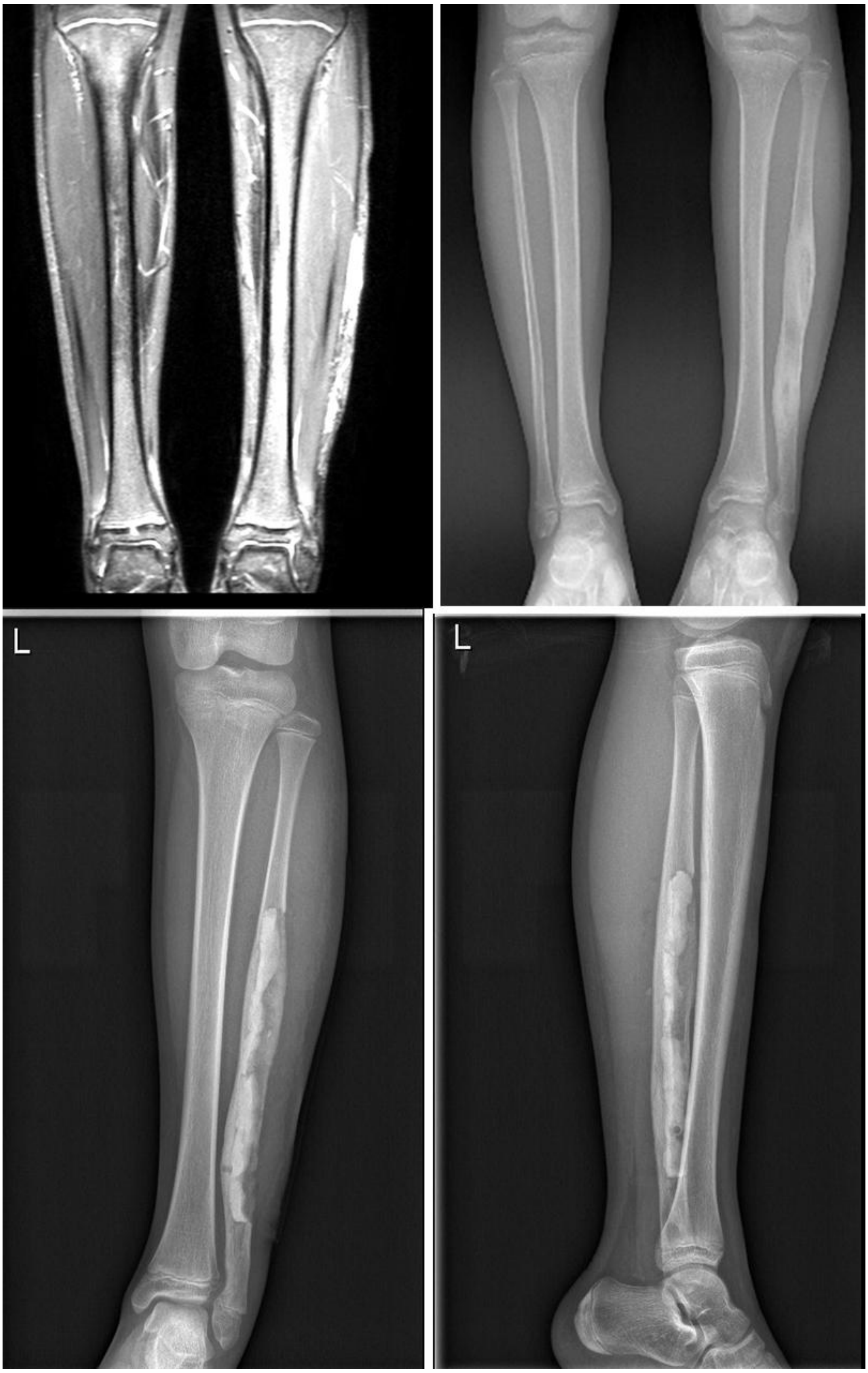

CASE 11. A 12 years old girl with a fibula osteomyelitis: preoperative X-ray and MRI, postoperative X-ray.

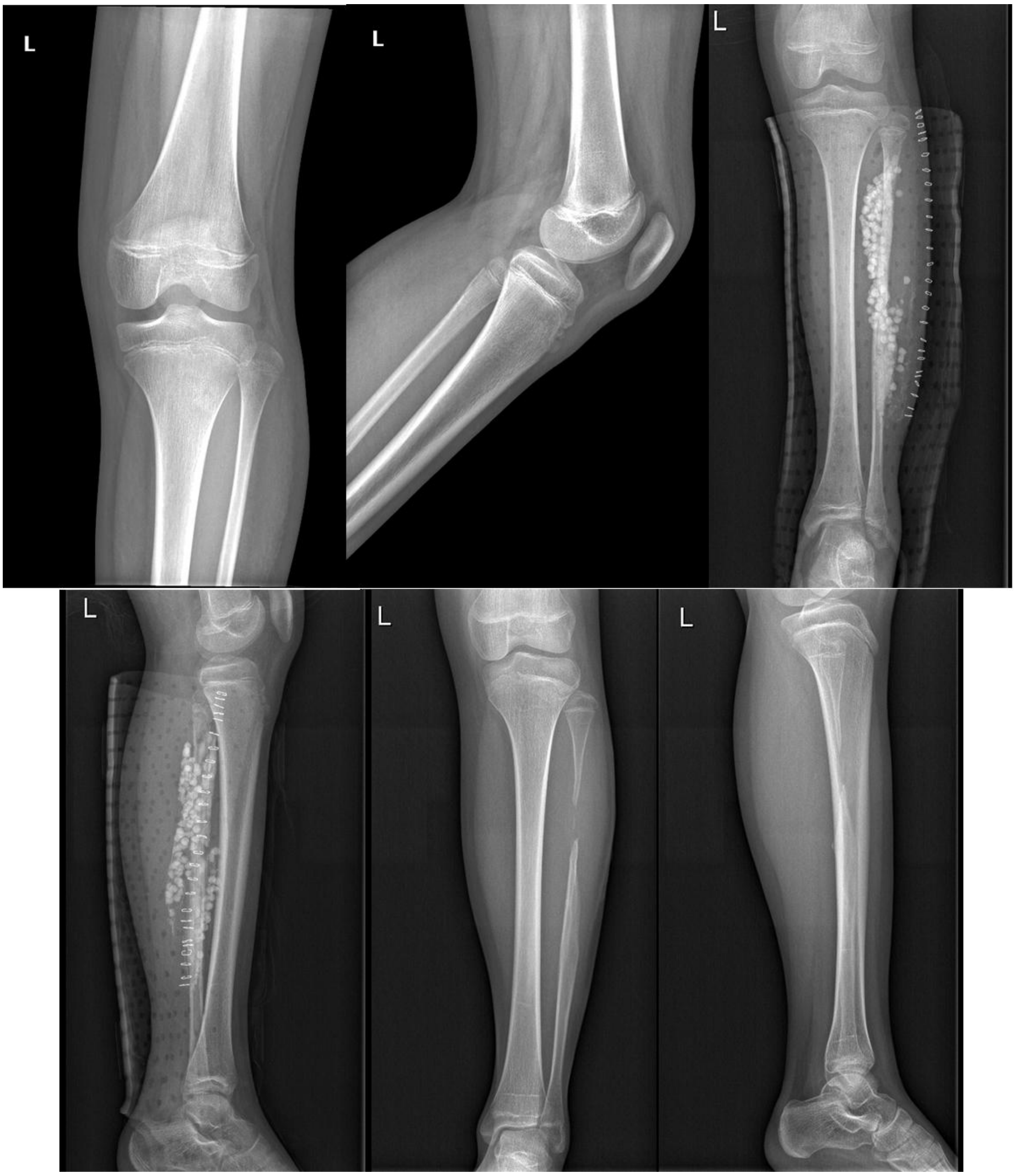

CASE 12. A 10 years old girl with a fibula osteomyelitis: preoperative X-ray, postoperative X-ray.

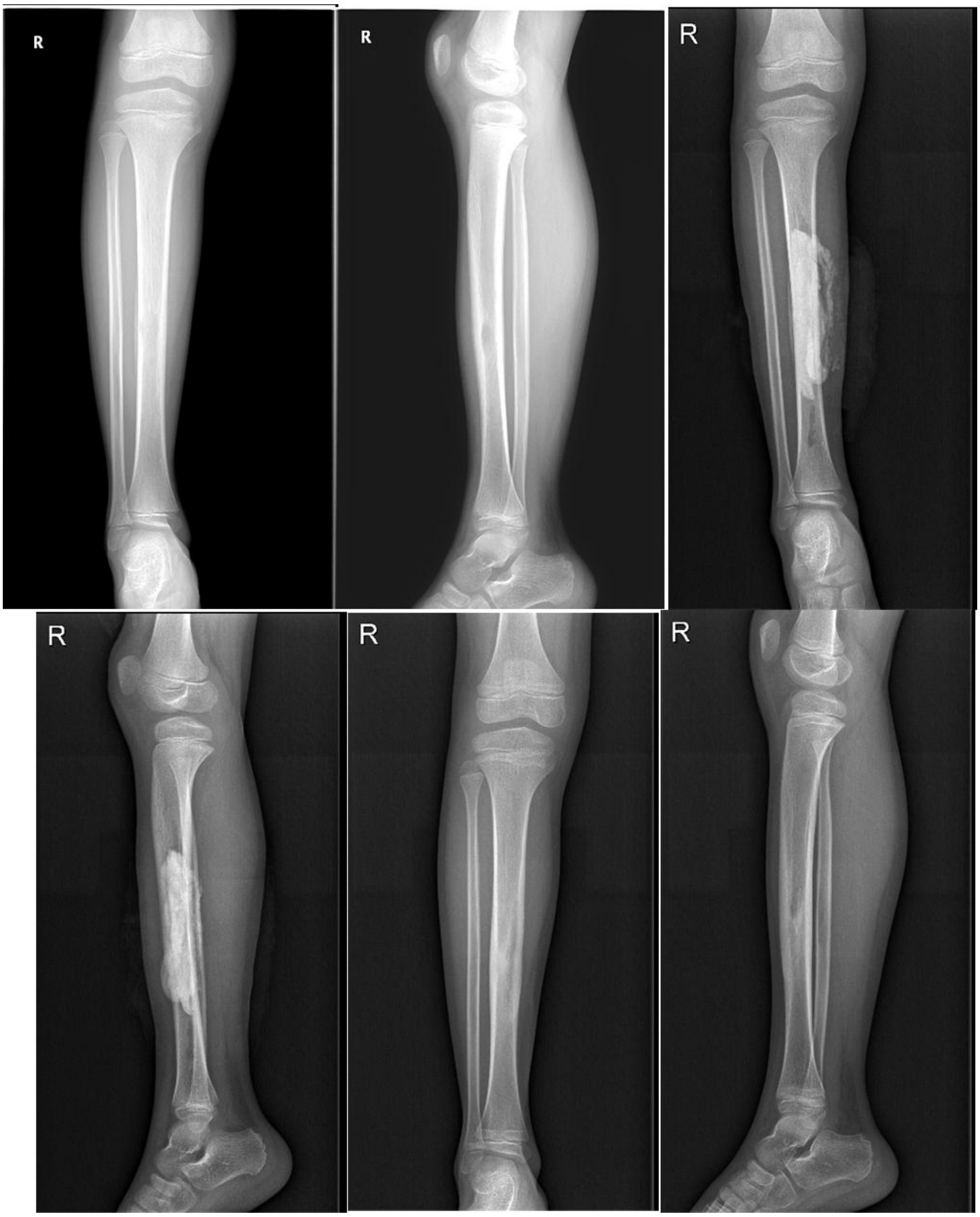

CASE 13. A 10 years old boy with a tibia osteomyelitis: preoperative X-ray, postoperative X-ray.

Reoperation

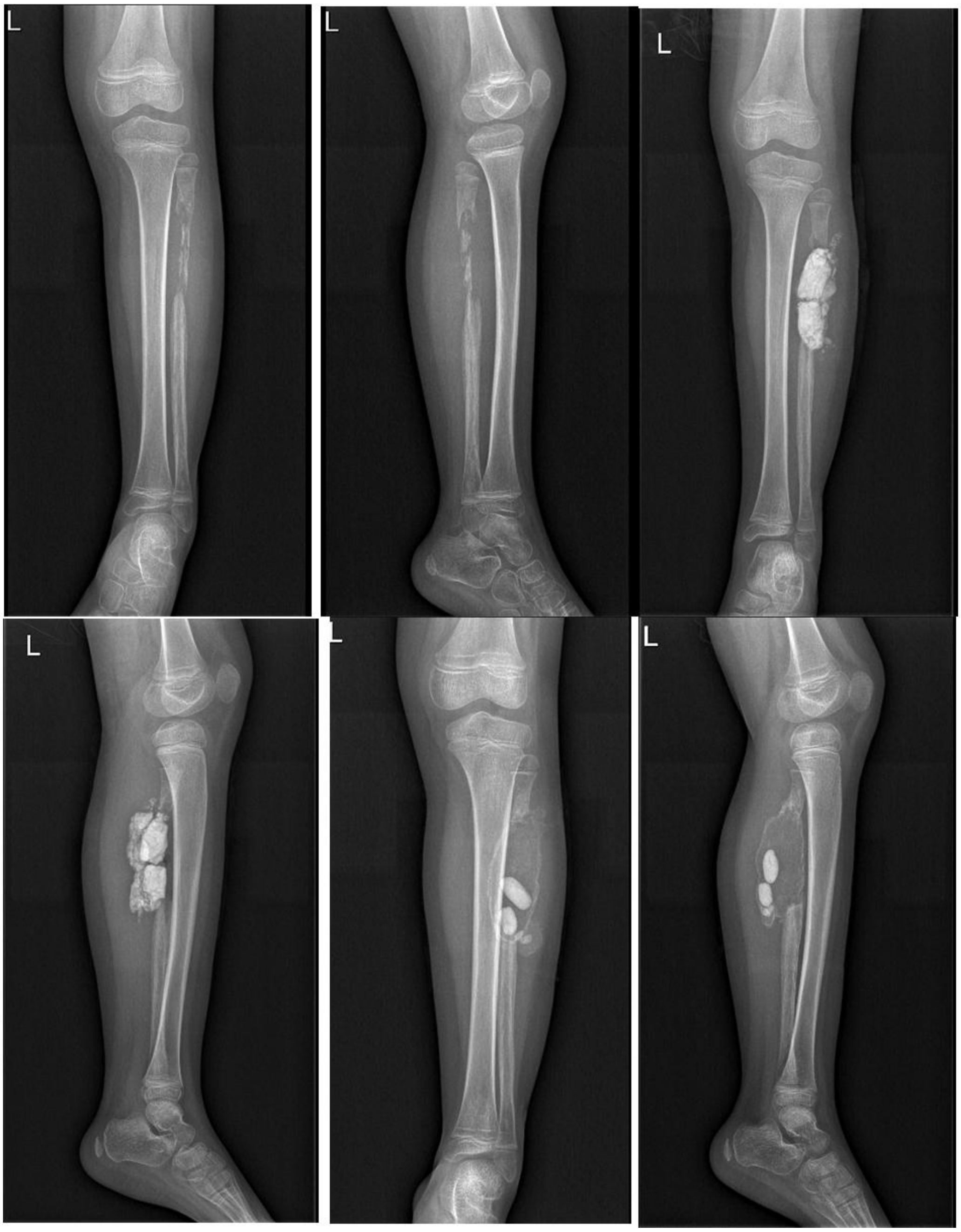

Case 1. A 6 years old girl with a fibula osteomyelitis: preoperative X-ray, postoperative X-ray.

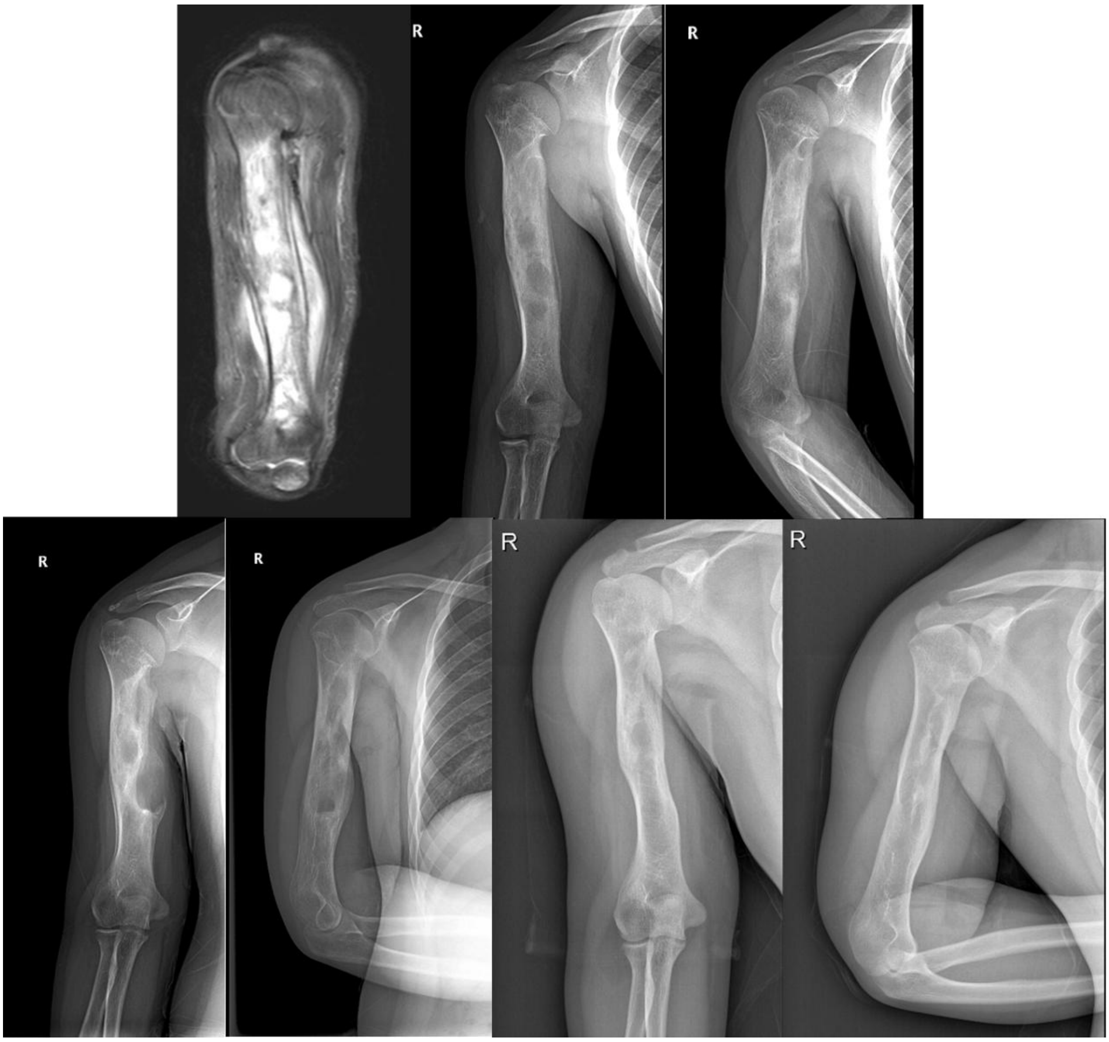

Case 2. A 14 years old boy with a humerus osteomyelitis: preoperative X-ray and MRI, postoperative X-ray.

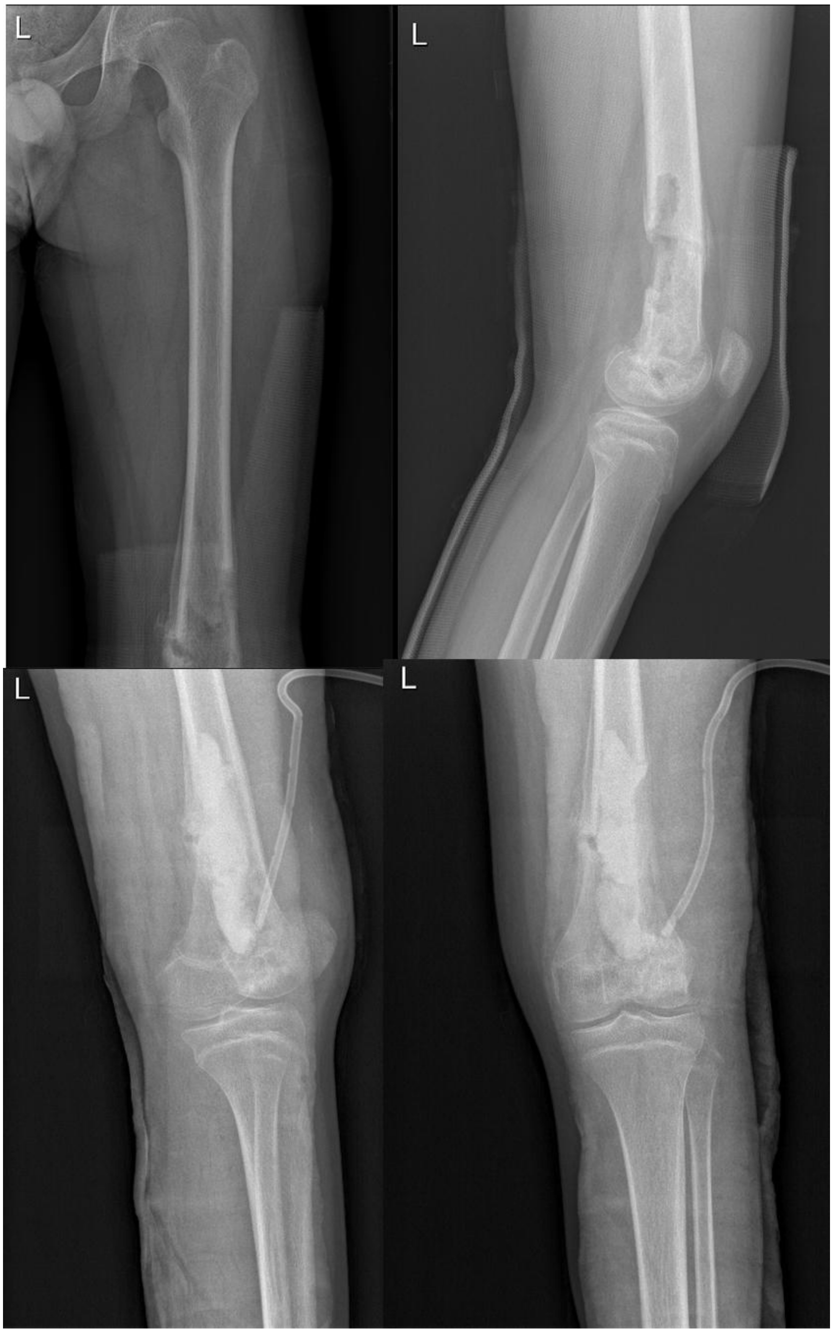

Case 3. A 15 years old boy with a femur osteomyelitis: preoperative X-ray, postoperative X-ray.

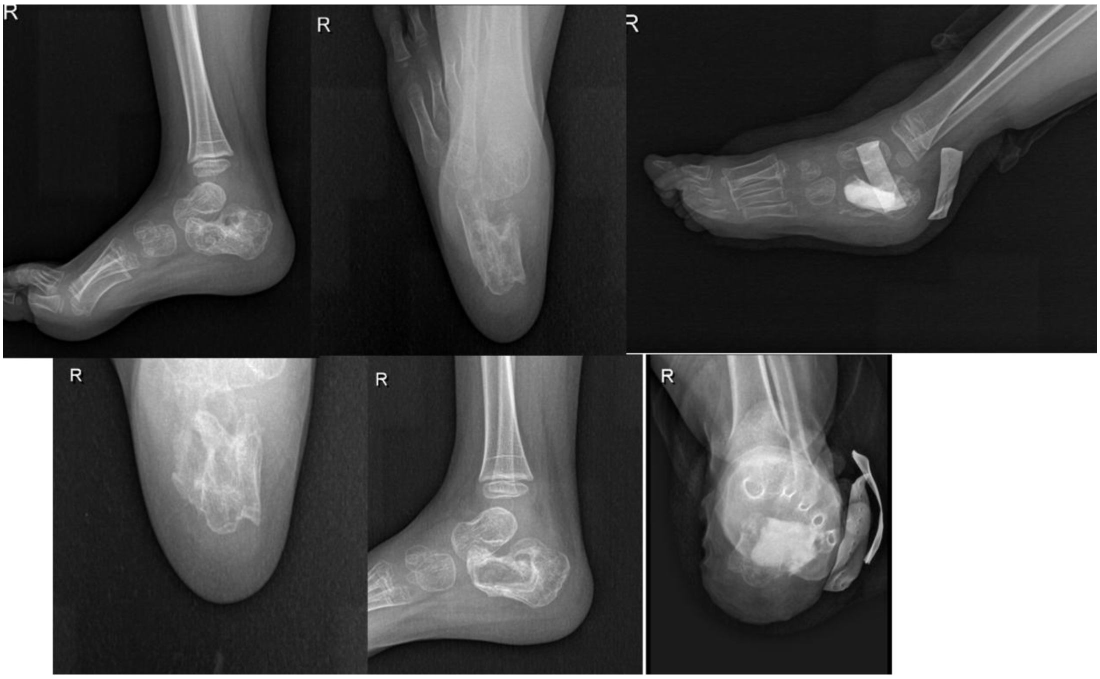

Case 4. A 3 years old boy with a calcaneus osteomyelitis: preoperative X-ray, postoperative X-ray.

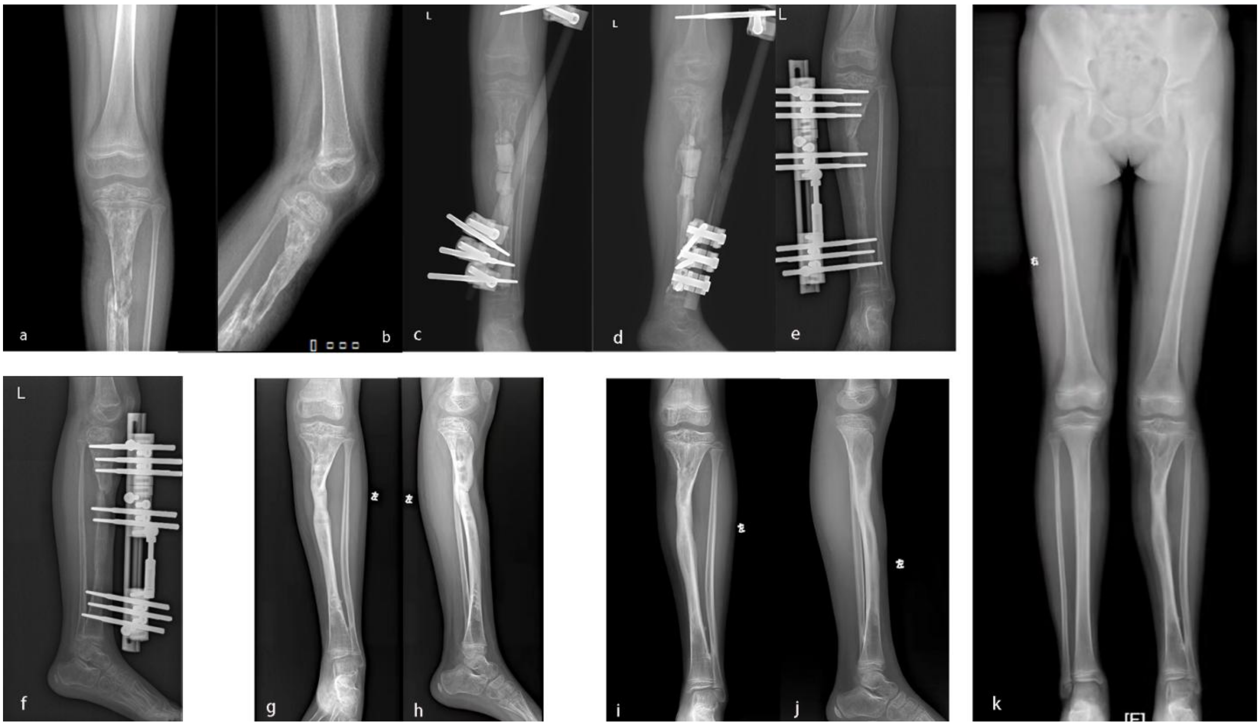

Case 5. A 6 years old boy with a tibia osteomyelitis: preoperative X-ray, postoperative X-ray. An external fixation was used for bone transport.

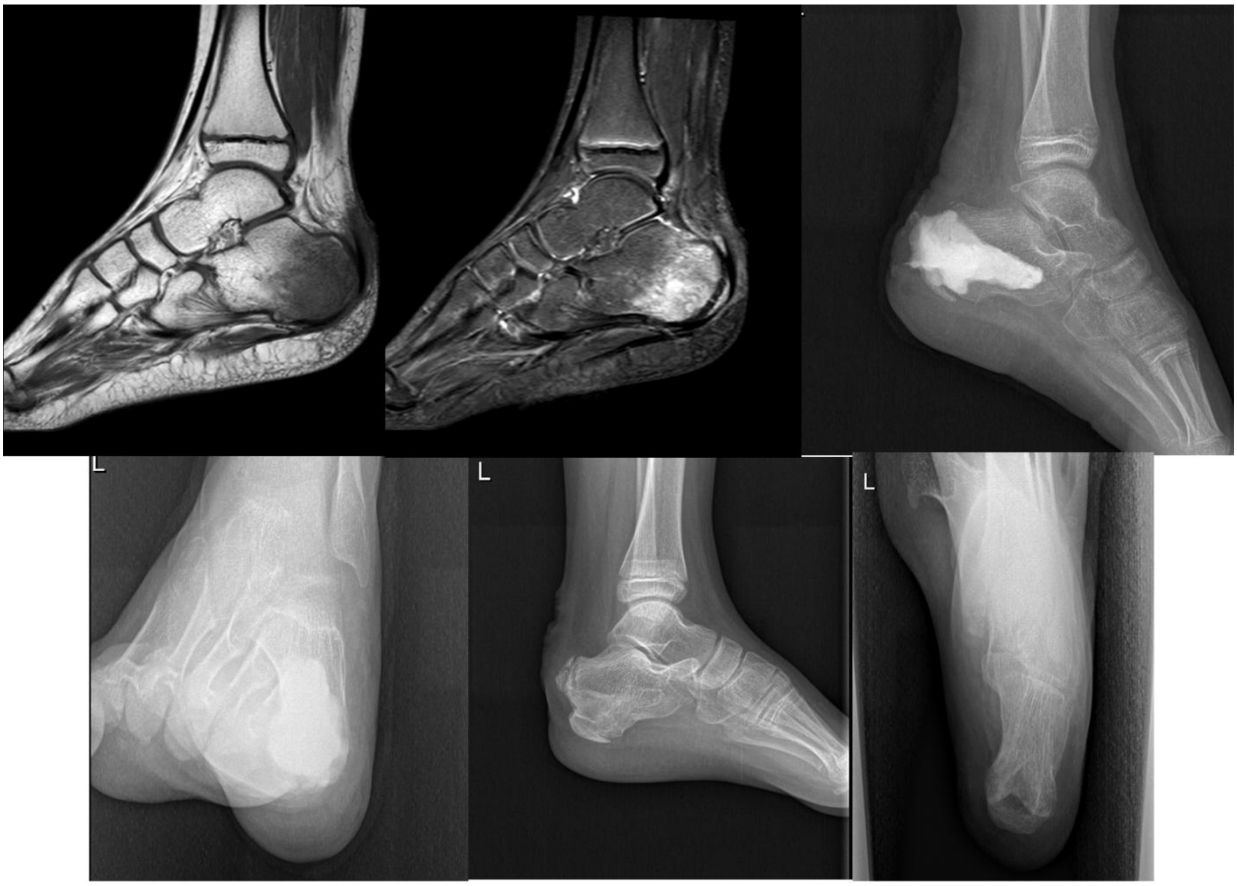

Case 6. A 10 years old boy with a calcaneus osteomyelitis: preoperative X-ray and MRI, postoperative X-ray.

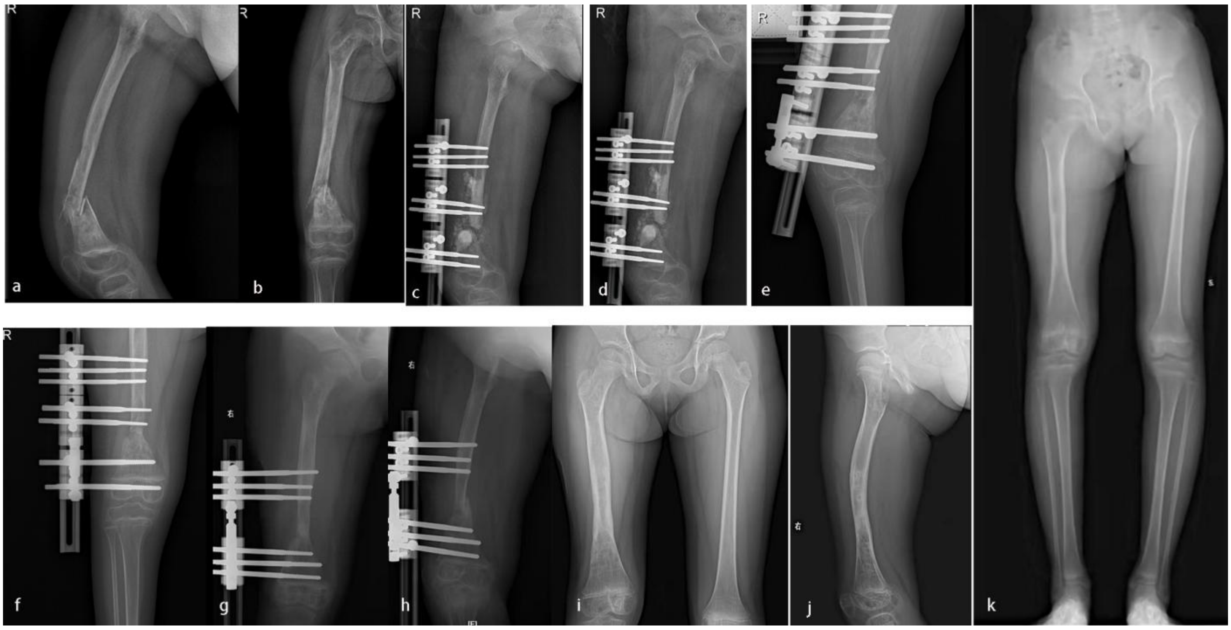

Case 7. A 5 years old girl with a femur osteomyelitis: preoperative X-ray, postoperative X-ray. An external fixation was used for bone transport.

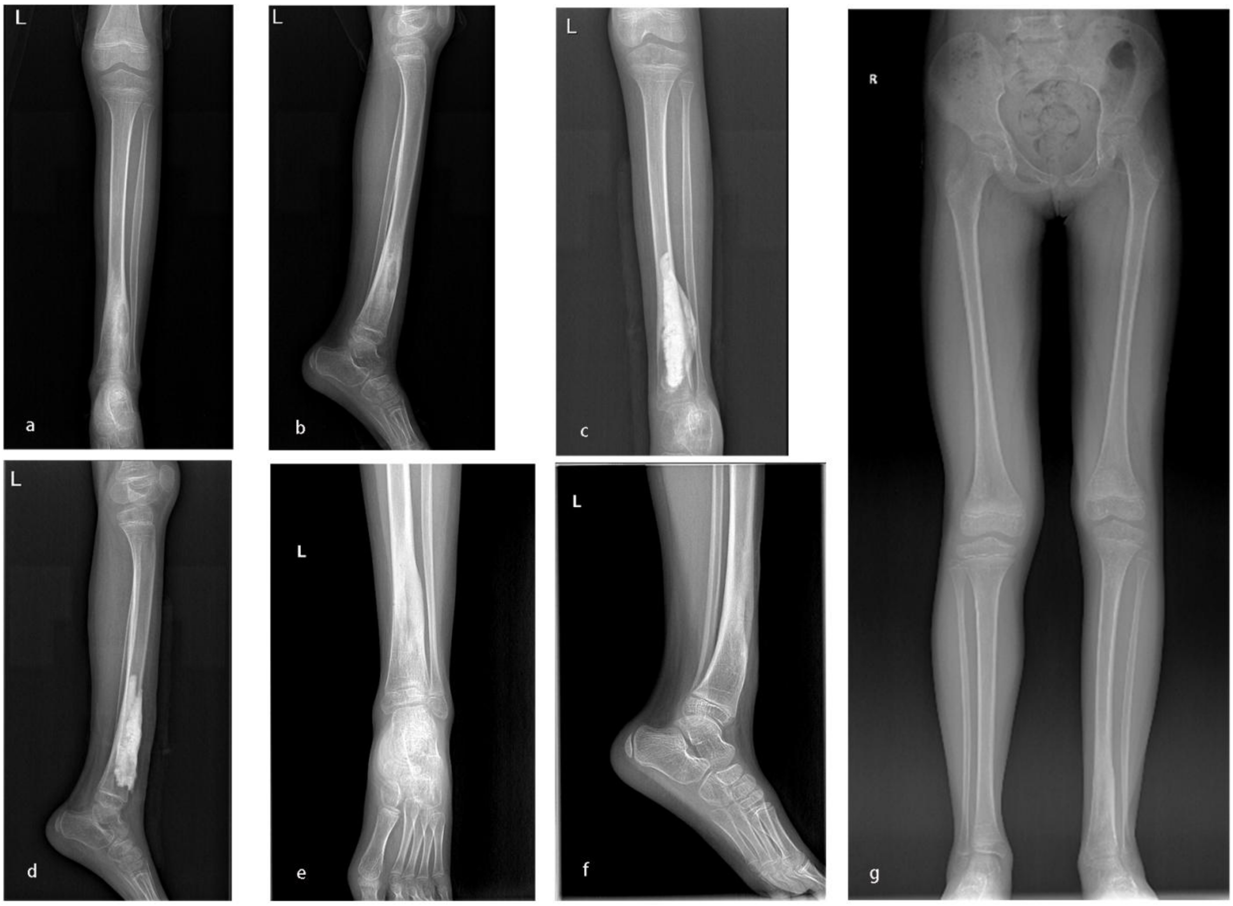

Case 8. A 7 years old boy with a tibia osteomyelitis: preoperative X-ray, postoperative X-ray.
